# Ethyl Acetate Extract from *Artemisia argyi* Prevents Liver Damage in ConA-Induced Immunological Liver Injury Mice via Bax/Bcl-2 and TLR4/MyD88/NF-*κ*B Signaling Pathways

**DOI:** 10.3390/molecules27227883

**Published:** 2022-11-15

**Authors:** Wenqian Yang, Fei Shao, Jiexin Wang, Tong Shen, Yu Zhao, Xueyan Fu, Liming Zhang, Hangying Li

**Affiliations:** 1 College of Pharmacy, Ningxia Medical University, Yinchuan 750004, China; 2 Ningxia Research Center of Modern Hui Medicine Engineering and Technology, Ningxia Medical University, Yinchuan 750000, China; 3 Key Laboratory of Ningxia Ethnomedicine Modernization, Ministry of Education, Ningxia Medical University, Yinchuan 750004, China

**Keywords:** *Artemisia argyi* Lévl. et Vant., phytochemistry, food therapy, dietary plant, immunological liver injury, Bax/Bcl-2 and TLR4/MyD88/NF-*κ*B

## Abstract

Background: Immunological liver injury (ILI) is a common liver disease and lacks potent drugs for treatment. *Artemisia argyi* Lévl. et Vant. (*A. argyi*), a medicinal and edible homologous plant usually used in diet therapy to cure various liver diseases, provides a great option for the prevention of ILI. Purpose: To investigate the effect that ethyl acetate extract of *A. argyi* (AaEA) on Concanavalin A (ConA)-induced ILI and the mechanism of regulating Bax/Bcl-2 and TLR4/MyD88/NF-*κ*B signaling pathways. Methods: The chemical components of AaEA were studied by LC-MS. In animal experiments, the positive control group was administrated diammonium glycyrrhizinate (DIG, 100 mg/kg), while different doses of AaEA groups (AaEA-H, AaEA-M, AaEA-L) were pretreated with AaEA 2.00, 1.00, and 0.50 g/kg, respectively, by intragastric for seven days, once every day. Then, ConA (12.00 mg/kg) was used through tail intravenous injection to establish the ILI model. The blood samples and livers were collected to test the degree of liver dysfunction, inflammation, oxidative stress, histopathological changes, and cell apoptosis. Real-time PCR and Western blotting analysis were used to explain the mechanism of regulating Bax/Bcl-2 and TLR4/MyD88/NF-*κ*B signaling pathways. Results: The way in which AaEA prevents liver damage in immunological liver injury (ILI) mice caused by ConA was investigated for the first time. Pretreatment with AaEA reduced the expression of ALT, AST, and inflammatory factors (TNF-*α* and IFN-*γ*). Meanwhile, AaEA also reduced MDA levels but upregulated the contents of IL-4, SOD, and GSH-px, alleviating oxidative stress induced by ILI. Western blotting and real-time PCR analysis demonstrated that AaEA could regulate the expression level and relative mRNA expression of key proteins on Bax/Bcl-2 and TLR4/MyD88/NF-*κ*B signaling pathways. Finally, 504 components from AaEA were identified by LC-MS analysis, mainly including flavones, phenolic acids, and terpenoids with anti-inflammatory and liver protective activities, which highlights the potential of AaEA for diet treatment of ILI. Conclusion: AaEA can work against ConA-induced ILI in mice by regulating Bax/Bcl-2 and TLR4/MyD88/NF-*κ*B signaling pathways, which has the potential to be a great strategy for the prevention of ILI.

## 1. Introduction

Immunological liver injury (ILI), a liver disease, is chronic and strongly linked to the immune system. The major characteristics of ILI are inflammatory cells infiltrating the liver, and the levels of transaminase and inflammatory factors significantly increase [1]. ILI is a key process that accelerates various liver disease progression, which may trigger and precede liver fibrosis, liver cirrhosis, and even hepatocarcinoma [2]. It is reported that the average incidence of ILI was 1.93 per 100,000 population from 2008 to 2016, and it showed an increasing trend year by year, which brought a huge burden to patients’ families and even society [3]. There is a lack of potent drugs for the treatment of ILI currently. Using medicinal and food homologous plants for health maintenance and food therapy is a traditional and useful method in China [4], which has drawn attention to the prevention of chronic liver disease and might be a new strategy for treating ILI.

The characteristics of immunological hepatic injury are immune dysfunction and unbalanced immune tolerance, which lead to inflammation storm. However, over-inflammation could damage host cells and lead to several immune diseases, even mortality [5]. As recognition receptors, Toll-like receptors (TLRs) occupy an important position in various immunological chronic diseases. TLR4, a member of the TLR family, has been one of the most widely investigated immune recognition receptors. It is reported that more than 20 ligands could activate the TLR4 pathway as TLR4 endogenous ligands. Then downstream adaptor proteins such as myeloid differentiation protein (MyD88) would be recruited. In addition, the recruitment is followed by a sequential cascade downstream to active AP-1, IRFs, NF-*κ*B, and STAT-1 signaling pathways, regulating proinflammatory cytokines release [6]. NF-*κ*B is a critical transcription factor in innate immune response as well as inflammation response, which regulates inflammatory cytokines to express [7]. After releasing inflammation factors (IL-4, TNF-*α*, and IFN-*γ*), the inflammation cascade would be triggered, and immune-related cells would be regulated. The cytokines accelerate the damage of liver tissue by influencing immune-related cell proliferation, differentiation, and apoptosis [8]. Lipopolysaccharide-induced acute liver injury was reported to be alleviated by emodin via TLR4 signaling pathway [9], and *Dendrobium officinale* polysaccharides were found to have an effect on acute liver injury caused by ethanol, the mechanism for which was related to TLR4/NF-*κ*B pathway [10]. The results provide a potential therapeutic approach to treating immune-mediated liver injury, which inhibits TLR4 and regulates MyD88, NF-*κ*B, and even other downstream molecules

*Artemisia Argyi* Lévl. et Vant. (*A. argyi*) is a medicinal and edible homology resource. Phytochemical and pharmacological studies found that the chemical components of *A. argyi* including flavonoid, terpenoid, and phenolic acids with antibacterial, anticancer, antitussive, expectorant, hemostatic, anticoagulant, and liver-protective activities [11]. As a popular dietary plant in China, *A. argyi* is usually cooked into glutinous rice cakes, dumplings, and meatballs for food therapy. Moreover, drinking *A. argyi* leaves tea is a unique healthy lifestyle in China that can strengthen the body and prevent diseases [12]. Dietary therapies and plant-derived active ingredients are receiving increasing attention in the treatment of liver diseases. For instance, green tea polyphenols were reported to possibly mitigate the plant lectins-induced liver inflammation and immunological reaction in C57BL/6 mice via NLRP3 and Nrf2 signaling pathways [13]. Studies have shown that *A. argyi* can regulate the cell cycle and downregulate the expression of cyclin D1 so that cell replication stops at the G_0_/G_1_ phase and cell proliferation is inhibited, thus achieving the effect of antiliver fibrosis [14]. In addition, *A. argyi* polysaccharide can prevent liver poisoning caused by acetaminophen, protect liver tissue cells, and thus achieve the purpose of liver protection [15]. Thus, *A. argyi* has the potential to prevent and treat liver disease.

In previous studies, we have explored food therapy for various chronic diseases [16,17], especially ILI. For example, the *n*-butanol part of *Viola yedoensis* could protect ILI mice [17]. During the discovery of dietary plants against ILI, we first found the ethyl acetate part of *A. argyi* (AaEA) possessed a significant efficiency in preventing ILI. In this study, the phytochemical composition of AaEA was analyzed at first. Second, the effects of AaEA on Concanavalin A (ConA)-induced ILI were evaluated in mice. Third, the mechanism by which AaEA counteracted the ConA-induced ILI by regulating Bax/Bcl-2 and TLR4/MyD88/NF-*κ*B pathways was investigated.

## 2. Results

### 2.1. LC-MS Analysis of AaEA

**Figure 1:**
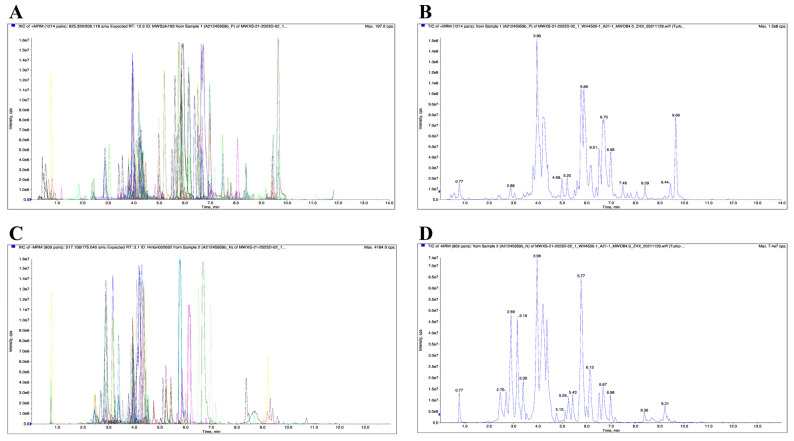
Chemical constituents of AaEA were analyzed by LC-MS. Positive ion chromatogram of AaEA (**A**,**B**) and negative ion chromatogram of AaEA (**C**,**D**).

**Figure 2:**
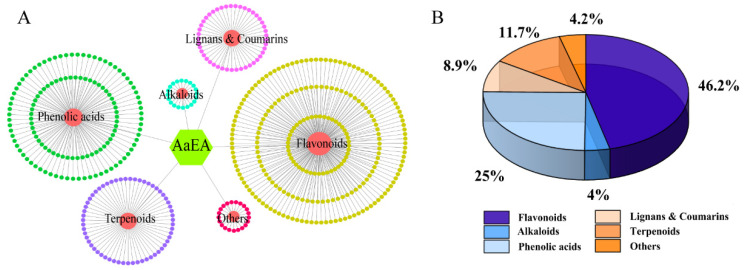
The types of compounds and their proportion in all compounds from AaEA. As shown in the diagram, the dark yellow spots stand for 233 flavonoids from AaEA, while green, purple, pink, blue, and red spots stand for 126 phenolic acids, 59 terpenoids, 45 lignans and coumarins, 20 alkaloids, and 21 others, respectively (**A**). The proportion of different component types (**B**).

As shown in Figure 1, helpful information about the molecular was provided by the ions of [M + H]^+^ and [M − H]^−^. The elements, molecules’ weight, and possible composition were compared with the local metabolic database. Finally, 504 components of AaEA were identified, including 233 flavones (46.2%), 20 alkaloids (4%), 45 lignans and coumarins (8.9%), 126 phenolic acids (25%), 59 terpenoids (11.7%), and 21 other compounds (4.2%) (Figure 2 and Supplementary Materials Tables S1–S6).

### 2.2. Effect of AaEA on ConA-Induced Liver Damage and Serum Transaminase Levels

**Figure 3:**
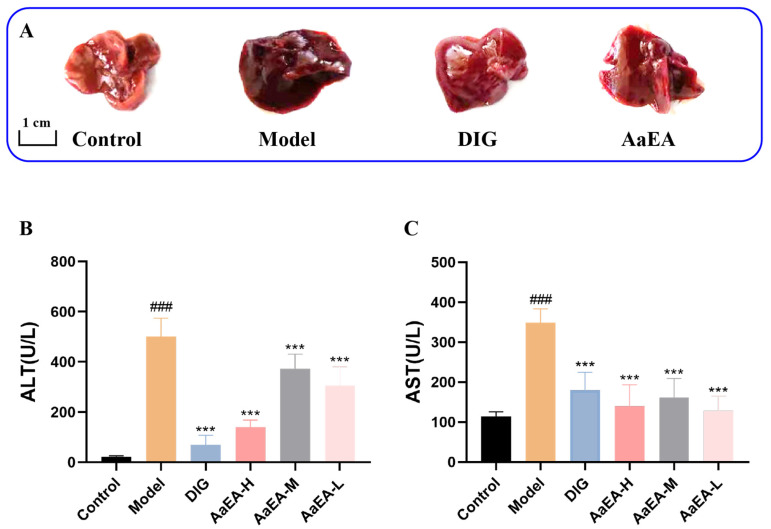
AaEA inhibited ConA-induced ILI in mice. Changes of liver tissues by the naked eye (**A**). Serum ALT activity (**B**) and AST activity (**C**) levels in different groups (n = 6). Unless indicated, no significant difference was observed between groups. Compared with control group, ^###^ *p* < 0.001. Compared with ConA group, *** *p* < 0.001.

Firstly, we preliminarily estimated the macroscopical character of the liver when the mice were sacrificed; the liver in the control group did not reveal any histological abnormalities, while the model group was found to have a dark red liver. The positive control group that was pretreated with diammonium glycyrrhizinate (DIG, 100 mg/kg) and AaEA pretreatment groups showed minor changes in liver color compared with the ILI mice (Figure 3A). Then, compared with the control group, markedly increased levels of ALT and AST were discovered in the ConA group (*p* < 0.001), while ALT and AST activities were lower in DIG and AaEA-pretreated groups compared with the ConA group (*p* < 0.001) (Figure 3B,C). The results demonstrated that animal models successfully established and pretreatment with AaEA could significantly prevent liver injury caused by ConA.

### 2.3. Effects of AaEA on Liver Oxidative Stress

**Figure 4:**
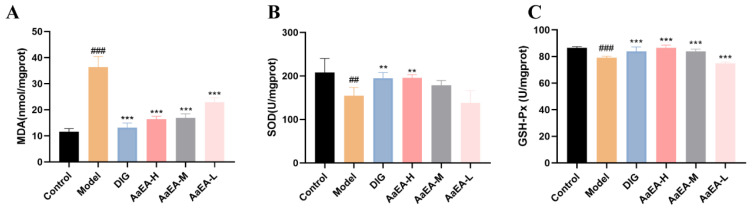
Effects of AaEA alleviated oxidative stress by regulating MDA level (**A**), SOD (**B**), and GSH-px activities (**C**) (n = 6). Unless indicated, no significant difference was observed between groups. Compared with control group, ^##^ *p* < 0.01 and ^###^ *p* < 0.001. Compared with ConA group, ** *p* < 0.01, and *** *p* < 0.001.

The MDA, GSH-px, and SOD activities were assayed to find out if AaEA has effects on alleviating oxidative stress caused by ILI. MDA was overproduced in the liver tissue of ILI mice in comparison to mice in the control group (*p* < 0.001), while the MDA activities were notably reduced in mice pretreated with DIG and AaEA, respectively (Figure 4A, *p* < 0.001). In the ConA group, activities of SOD (*p* < 0.01) and GSH-px (*p* < 0.001) decreased prominently compared to the control group, respectively. In contrast, pretreatment with DIG increased SOD levels (*p* < 0.01) as well as GSH-px activity (*p* < 0.001). Different doses of AaEA groups significantly improved GSH-px levels (*p* < 0.001), while only the AaEA-H group observably improved SOD levels (Figure 4B,C). All the results indicated that AaEA could alleviate oxidative stress caused by ILI.

### 2.4. Effects of AaEA on IL-4, TNF-α, and IFN-γ

**Figure 5:**
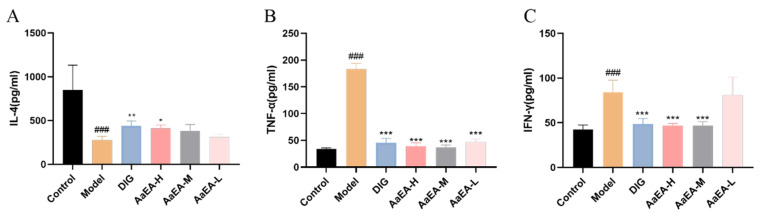
Effects of AaEA on IL-4 (**A**), TNF-*α* level (**B**), and IFN-*γ* (C) activities in liver tissues (n = 6). Unless indicated, no significant difference was observed between groups. Compared with the control group, ^###^ *p* < 0.001. Compared with model group, *** *p* < 0.001, ** *p* < 0.01, and * *p* < 0.05.

Inflammation and immune imbalance occupy an important position in the process of ILI. IL-4, TNF-*α*, and IFN-*γ* were tested to reveal the effects that AaEA is able to inhibit inflammation and regulate the imbalanced immune system. As shown in Figure 5, the levels of TNF-*α* and IFN-*γ* in the ConA group increased remarkably (*p* < 0.001) in comparison with the control group, respectively. However, compared to the ConA group, TNF-*α* expressions in the DIG group and different doses of AaEA groups decreased distinctly (*p* < 0.001). Similarly, the level of IFN-*γ* in DIG group was observably reduced (*p* < 0.001) as well as in AaEA-H and AaEA-M groups. Meanwhile, compared with the control group, the IL-4 level decreased significantly in ILI mice (*p* < 0.001). In contrast, the IL-4 levels of the DIG group and AaEA-H group were upregulated compared to the ConA group (*p* < 0.01, *p* < 0.05). The results cleared that AaEA had effects on inhibiting inflammation.

### 2.5. Histopathological Features of Mice Livers

**Figure 6:**
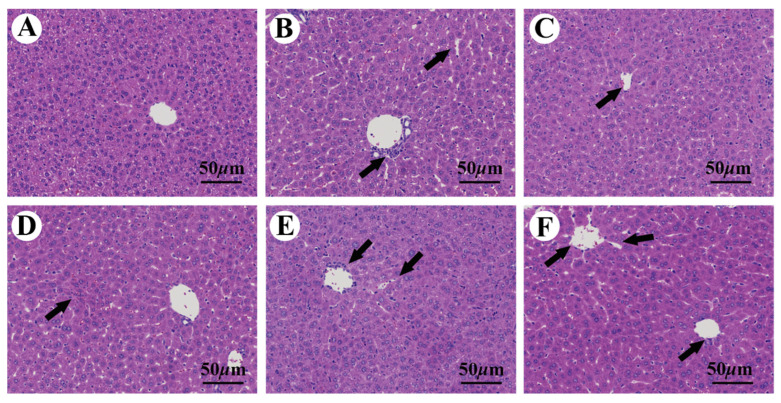
Images of liver damage stained by H&E (magnification: ×200). (**A**–**F**) represent the control, ConA, DIG, AaEA-L, AaEA-M, and AaEA-H groups, respectively. The cells’ disorder arrangement and infiltration of inflammatory cells are demonstrated by black arrows.

The liver slices were stained with H&E for histopathological analysis and to evaluate the positive effects of AaEA against ConA-induced ILI. As illustrated in Figure 6A, there was no histological abnormality in the control group animals, and normal hepatic cells with obvious hepatic sinusoids and visible central veins were recognized. In contrast, a histological liver section of model group mice showed variably sized cytoplasmic vacuoles with erythrocyte siltation. The inflammatory cells infiltration and the rupture of the hepatocyte nucleus were observed in the liver sections as well (Figure 6B). However, the DIG group and different doses of AaEA pretreatment groups reduced the disorganization of the hepatocytes and, in addition, decreased the infiltration of inflammatory cells. Compared to the ConA group, the normal architecture of hepatic cells could be observed in most areas of the liver section (Figure 6C−F).

### 2.6. Effects of AaEA on Liver Cell Apoptosis

**Figure 7:**
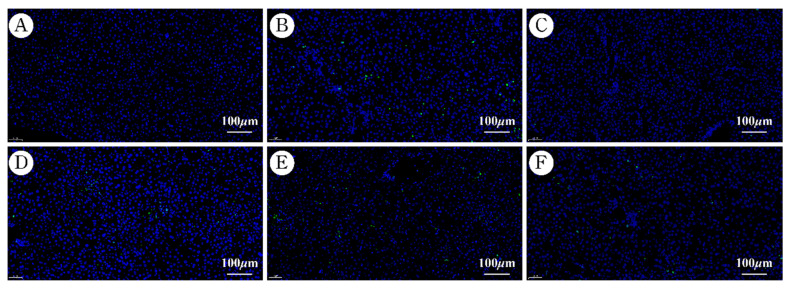
Effects of AaEA-mitigated liver cell apoptosis (magnification: ×20). (**A**–**F**) represent the control, ConA, DIG, AaEA-L, AaEA-M, and AaEA-H groups, respectively.

The apoptosis of liver cells was assayed by TUNEL staining. As shown in Figure 7, liver tissue apoptosis was aggravated since the liver injury. It can be seen that the apoptosis of liver tissue was slight in the DIG and AaEA groups in comparison with that in the ConA group, respectively (Figure 7C−F). The results illustrate that AaEA could mitigate the apoptosis of liver tissue cells.

### 2.7. AaEA Inhibited the Bax/Bcl-2 and TLR4/MyD88/NF-κB Signaling Pathways

According to the results of the pharmacodynamics study, the high-dose AaEA (AaEA-H) group had the best effect on ConA-induced ILI. Hence, the AaEA-H group was used for the mechanism study. TLR4/MyD88 and its downstream signaling pathways are directly linked to a sequential cascade and involved in various biological processes. The NF-*κ*B pathway plays a critical role in inflammation. The Bax/Bcl-2 signaling pathway is closely related to apoptosis. Regulating key proteins on Bax/Bcl-2 and TLR4/MyD88/NF-*κ*B pathways is considered a potential therapy or preventive strategy for ILI.

**Figure 8:**
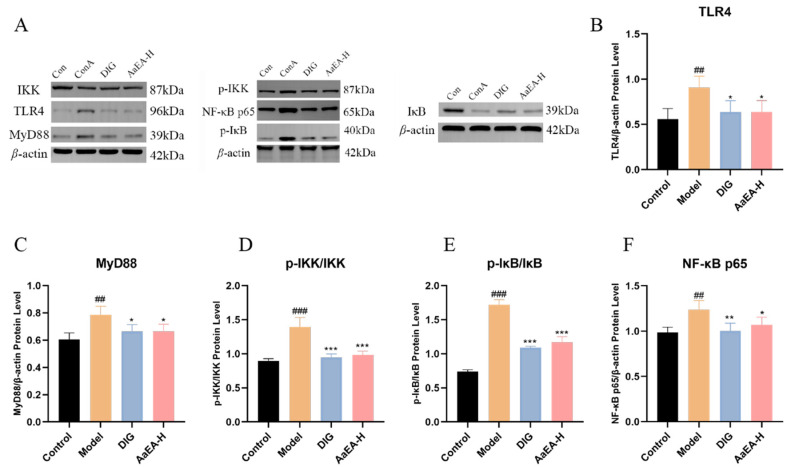
Effects of AaEA-H on regulating the key proteins of the TLR4/MyD88/NF-*κ*B pathway (n = 3). Western blot analysis of protein expressions of TLR4, MyD88, p-I*κ*B, I*κ*B, p-IKK, IKK, and NF-*κ*B p65 (**A**). TLR4 level normalized with *β*-Actin (**B**). MyD88 level normalized with *β*-Actin (**C**). The ratio of p-IKK protein expression level to IKK protein expression level (**D**). The ratio of p-I*κ*B protein expression level to I*κ*B protein expression level (**E**). NF-*κ*B p65 level normalized with *β*-Actin (**F**). Unless indicated, no significant difference was observed between groups. Compared with control group, ^##^ *p* < 0.01 and ^###^ *p* < 0.001. Compared with ConA group, * *p* < 0.05, ** *p* < 0.01 and *** *p* < 0.001.

**Figure 9:**
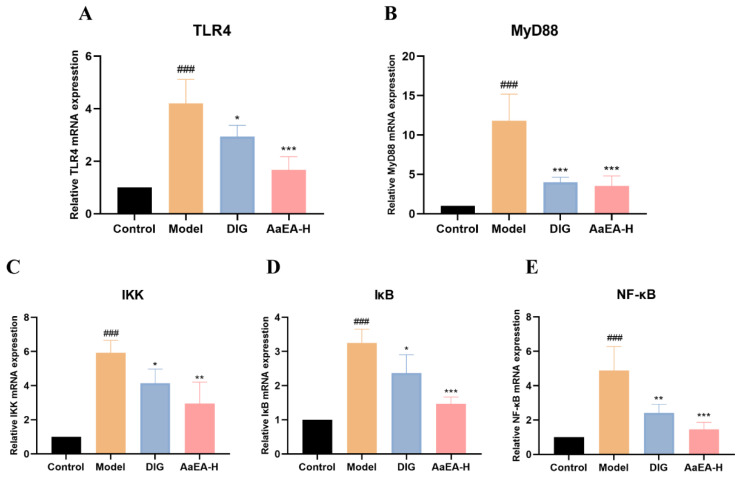
Effects of AaEA-H on each relative target gene of the TLR4/MyD88/NF-*κ*B pathway (n = 3). Relative TLR4 mRNA expression level in other groups compared with the control group (**A**). Relative MyD88 mRNA expression level (**B**). Relative IKK mRNA expression level (**C**). Relative I*κ*B mRNA expression level (**D**). Relative NF-*κ*B P65 mRNA expression level (**E**). Unless indicated, no significant difference was observed between groups. Compared with control group, ^###^ *p* < 0.001. Compared with model group, * *p* < 0.05, ** *p* < 0.01, and *** *p* < 0.001.

The key proteins and relative mRNA expression on Bax/Bcl-2 and TLR4/MyD88/NF-*κ*B pathways were measured by Western blotting and real-time PCR technology (Figure 8 and Figure 9). As shown in Figure 8, compared with mice in the control group, the protein and mRNA expression of TLR4, MyD88, p-I*κ*B, p-IKK, and nuclear NF-*κ*B were remarkably elevated in ConA group, and expressions of I*κ*B and IKK were observably declined. Compared with the ConA group, TLR4, MyD88, p-I*κ*B, p-IKK, and nuclear NF-*κ*B were downregulated in the DIG group and AaEA-H pretreatment group. The results of real-time PCR showed that relative mRNA of TLR4, MyD88, p-I*κ*B, p-IKK, and nuclear NF-*κ*B were upregulated in the model group while being downregulated in the DIG and AaEA-H group (Figure 9). All the data indicated that pretreated with AaEA could inhibit the TLR4/MyD88/NF-*κ*B signaling pathway.

**Figure 10:**
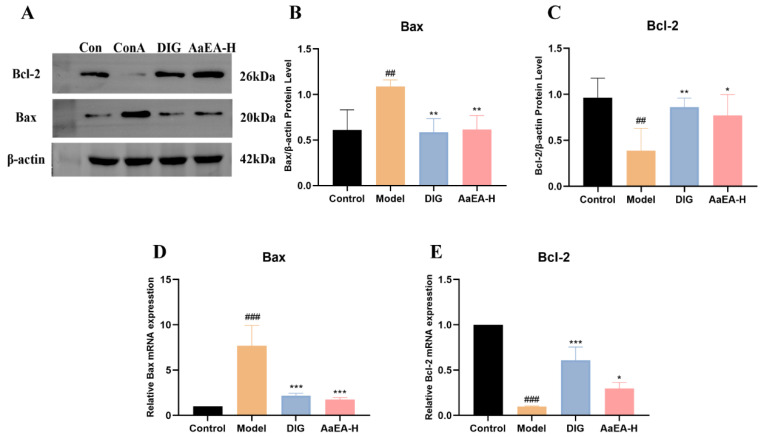
Effects of AaEA-H on Bax and Bcl-2 (**A**). Statistical analysis of Bax and Bcl-2 protein and mRNA relative expressions (**B**–**E**) (n = 3). Unless indicated, no significant difference was observed between groups. Compared with control group, ^##^ *p* < 0.01 and ^###^ *p* < 0.001. Compared with ConA group, * *p* < 0.05, ** *p* < 0.01, and *** *p* < 0.001.

Bax/Bcl-2 occupies a crucial position in apoptosis. It was observed that Bax expression was enhanced significantly and Bcl-2 expression decreased dramatically after the occurrence of liver injury (*p* < 0.001) (Figure 10B,C), which indicated that in comparison with the control group the apoptosis was aggravated in the ConA group. Then AaEA-H pretreatment group reduced Bax expression and improved Bcl-2 expression, respectively (*p* < 0.001), when compared with the ConA group, alleviating ILI-caused cell apoptosis (Figure 10B,C). The analysis of mRNA expression showed the same results (Figure 10D,E).

## 3. Materials and Methods

### 3.1. Plant Material

The *A. argyi* was herborized from Xiji County, Ningxia, China (105°38′–105°81′ E, 35°54′–35°95′ N). The plant (NO. 20210323) was identified by Professor Xueyan Fu according to the China Pharmacopoeia. The sample was deposited in the third category of traditional Chinese medicine extraction and separation, Ningxia Engineering and Technology Research Center for Modernization of Chinese Medicine, Yinchuan, Ningxia.

### 3.2. Sample Preparation

The overground parts of *A. argyi* were dried and cut into small fragments. Then the material was extracted with 95% ethyl alcohol three times, and all extract solvents were evaporated under a vacuum. Then the extract was gained and partitioned three times with ethyl acetate and normal butyl alcohol, respectively (1 g of the ethyl acetate extract is equivalent to 25 g of *A. argyi*). Part of the ethyl acetate extract was used for chemical components analysis by a liquid chromatograph-mass spectrometer (LC-MS), and the other part was totally dried and yielded the remedy powder for in vivo assay.

### 3.3. LC-MS Analysis

The components of AaEA were identified by using the method reported before [18]. In brief, a UPLC-ESI-MS/MS system (UPLC, SHIMADZU Nexera X2, MS, Applied Biosystems 4500 Q TRAP) with an Agilent SB-C18 column (1.8 µm, 2.1 mm × 100 mm) was used. The linear gradient was 5% A (pure water with 0.1% formic acid) and 95% B (acetonitrile with 0.1% formic acid) to a composition of 95% A and 5.0% B. The effluent was alternatively connected to Q TRAP-MS (source temperature 550 °C; ion spray voltage 5500 V (positive ion mode)/−4500 V (negative ion mode).

### 3.4. Animals and Experimental Design

Obtained from the Animal Experimental Center of Ningxia Medical University (SCXK2020-0001, 8 September 2019) seventy-two ICR mice (half male, 18−22 g) were raised in an SPF laboratory at Ningxia Medical University, in accordance with the guidelines and ethics regulations of the Chinese Council for Animal Care.

**Figure 11:**
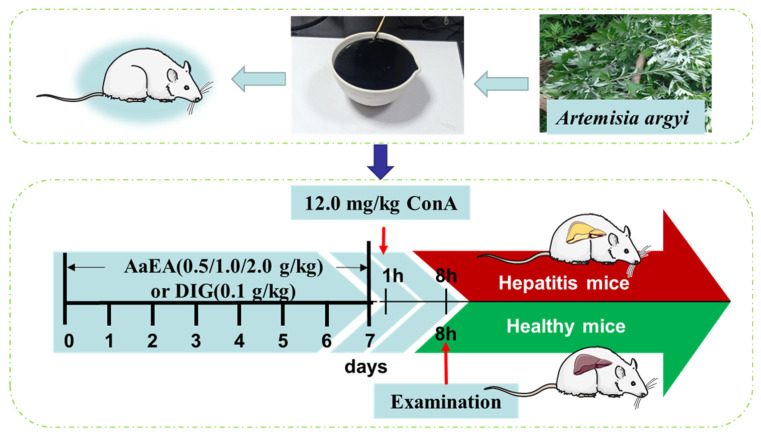
The process is designed for animal experiments.

As shown in Figure 11, after 3 days of adaptive feeding, the animals were separated into six groups (n = 12) randomly. The drugs were administrated intragastrically for seven days, once every day. The control and model groups received carboxyl methyl cellulose (CMC-Na), and the positive control group was administrated diammonium glycyrrhizinate (DIG, 100 mg/kg). Different doses of AaEA groups (AaEA-H, AaEA-M, AaEA-L) were pretreated with AaEA 2.00, 1.00, and 0.50 g/kg, respectively. After an hour after the last intragastric, the other groups received ConA (12.00 mg/kg) through tail intravenous injection to establish the ILI model, except for the control group. The eyeball extirpating method was used to collect fresh blood samples 8 h later. The animals were sacrificed by cervical dislocation, and their livers were collected.

### 3.5. Serum Biomarker Assays

The ALT and AST activities were assayed using the assay kit (Jiancheng Bioengineering Institute, Nanjing, China), respectively, following the protocol of the manufacturer. The results were expressed as an international unit (U/L).

### 3.6. Oxidative Stress-Related Factors

The supernatant of liver tissue was obtained after centrifugation (12,000× *g*, 4 °C, 10 min) after freezing and grinding. MDA, GSH-px, and SOD levels were measured with corresponding assay kits (Jiancheng Bioengineering Institute), respectively. The content of MDA was expressed as nmol per milligram protein. GSH-px and SOD contents were expressed as U per milligram protein.

### 3.7. Inflammatory Factors

ELISA kits (Elabscience Biotechnology, Wuhan, China) were used to measure the levels of hepatic IL-4, TNF-*α,* and IFN-*γ*, following the protocol for the users. Absorbance was measured at 450 nm.

### 3.8. Histopathological Analysis

The liver samples were fixed in 4% paraformaldehyde (PA), embedded in paraffin, and sliced into 5 μm slices. The slices were stained with hematoxylin and eosin (H&E). Pannoramic MIDI and 3DHISTCH were used to evaluate liver morphology.

### 3.9. TdT-Mediated dUTP Nick-End Labeling (TUNEL) Staining

Briefly, liver sections were deparaffinized, then permeabilized with 0.2% Triton X-100 and stained with 50 μM TUNEL reagent, respectively. Next, 4′-6-diamino-2-phenylindole was used to stain the cell nuclei. A fluorescence microscope was used to observe the results.

### 3.10. Real-Time PCR Assay

Total liver tissue RNA was extracted by the Total RNA kit. Then a PrimeScript RT Master Mix kit was used to transcribe 1 μg of the total RNA to cDNA. Gene expression was examined by SYBR Green Ⅰ fluorescence method on an ABI PRISM 7700 sequence detection system, referring to the method reported previously [19]. The primers used in the study are listed in Supplementary Materials Table S7. The relative expression of each target gene was quantified by the 2^−ΔΔCt^ method.

### 3.11. Western Blotting Analysis

Referring to methods in previous studies [20], WB analysis was carried out to investigate the relative expression of major proteins on Bax/Bcl-2 and TLR4/MyD88/NF-*κ*B pathways. The primary antibody included TLR4 (1:1000), MyD88 (1:1000), IKK (1:2000), p-IKK (1:1000), I*κ*B (1:2000), p-I*κ*B (1:1000), NF-*κ*B p65 (1:1000), Bax (1:1000), Bcl-2 (1:1000), and *β*-actin (1:5000). The secondary antibodies included goat antirabbit IgG H&L (HRP, 1:5000) and goat antimouse IgG H&L (HRP, 1:5000).

### 3.12. Statistical Analysis

All data were presented using the mean ± standard deviations (SD) in this study. The one-way analysis of variance (ANOVA) followed by Duncan’s multiple range test was used to determine the statistical difference among three or more groups. *p* < 0.05 was considered statistically significant. GraphPad Prism 8 software (San Diego, CA, USA) was used to analyze and visualize the results.

## 4. Conclusions and Discussion

The exact etiology and pathogenesis of ILI are complex and remain unknown. A lot of innate immune response cells are activated first and stimulated by endogenous or exogenous antigens in the liver. Then inflammatory factors are released and invade the liver tissue. Next, an excessive inflammatory response, as well as a strong immune response, occur, and the immune tolerance mechanism is destroyed, resulting in liver cell necrosis and apoptosis of liver tissue damage [21]. The production of free radicals and antioxidants are significantly reduced at the same time, causing the lipid peroxidation chain reaction of tissue cells, severe oxidative stress reaction, and initiation of cell apoptosis. Eventually, liver parenchyma injury is directly or indirectly caused [22]. Therefore, reducing the inflammatory response and oxidative stress to alleviating liver injury is a very promising therapeutic approach for immune liver injury [23].

The ConA-induced liver injury mouse model was successfully established to induce acute liver injury for the first time in 1992 [24], which resembles immune liver injury in humans and provides an ideal tool for further study of molecular mechanisms and screening therapeutic drugs. In this study, compared to the control group, liver tissues in ILI mice had visible changes in color and size (Figure 3A). The ALT and AST in ILI mice also climbed sharply when compared with the control group (Figure 3B,C). The inflammation generation and liver damage were notably demonstrated in the histological analysis of HE-stained liver sections (Figure 6A,B), which illustrated the ConA-induced ILI model was established successfully. As expected, AaEA demonstrated a prominent effect on liver protection, which was presented by normalizing liver pathological changes and serum levels of ALT and AST (Figure 3 and Figure 6D−F).

An increasing amount of evidence certifies that inflammation and oxidative stress are crucial in liver disease pathogenesis [25], culminating in cell apoptosis and liver fibrosis. Under normal conditions, Th1/Th2 cells, a subset of helper T lymphocytes, are in equilibrium. In the ConA-induced hepatitis model, Th1/Th2 was imbalanced and changed to the Th1 or Th2 state. In Figure 5A, the level of IL-4, a Th2 cytokine, was downregulated in the ConA group compared with the control group, while it was upregulated in the DIG group and AaEA-H group. Two Th1 cytokines, TNF-*α* and IFN-*γ,* are also proinflammatory cytokines, which were expressed at higher levels in the ConA group and downregulated in medication administration groups (Figure 5B,C). The results indicated that a Th1/Th2 imbalance existed in the model group, which was the same as reported [26]. We also found that AaEA could regulate the imbalance significantly. The levels of SOD, MDA, and GSH-px are critical parameters to reflect the potential antioxidant activity as well as the oxidative damage in the liver [27]. Our findings showed that AaEA significantly inhibited the inflammation in livers by reducing the excessive TNF-*α* and IFN-*γ* production (Figure 5). Furthermore, the oxidative stress was alleviated by decreasing the MDA level (Figure 4A), increasing the level of SOD and GSH-px in the AaEA pretreatment group (Figure 4B,C). Based on all the data in this study, it is concluded that AaEA intervention could alleviate inflammatory response and oxidative stress against ILI.

The chemical composition of *A. argyi* was identified in this study, including organic acids [28], flavonoids [28], terpenoids [29], and polysaccharides. Several kinds of phytochemicals in *A. argyi* were clarified to confer health benefits. For instance, the crude extract, flavonoids, phenolic acids, and terpenoids were reported to have an effect of immunosuppressive [30], antioxidant [31], and anti-inflammatory [32] activities, respectively. The analysis of LC-MS in this study demonstrated that 504 components of AaEA were identified, including flavones, alkaloids, lignans and coumarins, phenolic acids, terpenoids, and other compounds (Figure 2 and Supplementary Materials Tables S1–S6). As a result, the bioactivities of such components from AaEA are considered to have an effect on ConA-induced ILI accompanied by inflammation and oxidative stress.

Overactivation of Toll-like receptors (TLRs) signals can lead to severe immune-related diseases. TLR4 is the receptor of lipopolysaccharide (LPS). After TLR4 received extracellular stimulation (LPS), adaptor protein myeloid differentiation primary response protein (MyD88) was necessary to transmit signals downward [33] and activated a sequential cascade downstream, including the inhibitor of nuclear factor kappa-B kinase (IKKs) phosphorylation and ubiquitination, the inhibitor of NF-*κ*B (I*κ*B) proteolytic degradation, and activation and translocation of downstream transcription factor nuclear factor Kappa (NF-*κ*B). The NF-*κ*B signaling pathway is a critical intracellular signaling pathway, mediating the transcription of target genes and initiation of the immune response [34]. The inflammatory cells would infiltrate in the liver, mediating the organism’s natural immune defense. As shown in Figure 8B and Figure 9A, the protein expression and relative mRNA expression of TLR4 were observably increased in the ConA group, which indicated that the TLR4 signaling pathway was activated. The protein and relative mRNA expression of MyD88 was measured and increased significantly in model mice (Figure 8C and Figure 9B), which cleared that the MyD88-dependent signaling pathway was involved in a ConA-induced inflammation response. The expression of p-IKK/IKK and p-I*κ*B/I*κ*B (Figure 8D,E), as well as their relative mRNA expression (Figure 9C,D), were overexpressed in model mice, of which the meaning is that a sequential cascade downstream was activated, and the inflammation responses were magnified. Finally, the nuclear NF-*κ*B expression was increased in ConA group (Figure 8F and Figure 9E), indicating the downstream NF-*κ*B was activated, and proinflammatory cytokines were promoted. Inflammation and oxidative stress associated with liver injury promoted liver cell apoptosis. Apoptosis was one major cause of liver injury at the same time [19]. The expression of Bcl-2-Associated X Protein (Bax) was to promote apoptosis, while B-cell lymphoma-2 (Bcl-2) was expressed to inhibit apoptosis [35]. As shown in Figure 7, apoptosis was aggravated after the occurrence of ILI. The Bcl-2 expression was reduced signally, while Bax was overexpressed (Figure 10). Our findings demonstrated that apoptosis was closely related to liver damage in ILI. In addition, there was no toxicity in any of the three doses (2.00, 1.00, and 0.50 g/kg) used in our animal experiments, and all the doses were within the safe concentration range. According to the previous study of acute oral toxicity test, the LD_50_ of *A. argyi* ethanol extract was more than 20 g/kg so that there would not be apoptosis caused by the cytotoxic of AaEA [36]. As expected, this decreased levels of TLR4, MyD88, IKK, I*κ*B, NF-*κ*B, and Bax, while a raised level of Bcl-2 was shown in the AaEA-H group (Figure 8, Figure 9 and Figure 10). The above data and declined proinflammatory factors levels illustrate that AaEA-H intervention protected against ConA-induced ILI via Bax/Bcl-2 and TLR4/MyD88/NF-*κ*B signaling pathways.

There are still many aspects to explore in the future. For instance, lots of evidence indicated that NKT cells played a very important role in the ConA-induced hepatitis model, bridging innate and acquired immunity [37]. Other researchers found that neutrophile granulocyte macrophages and eosinophils participated in the ConA-induced hepatitis model as well [38]. It is not clear whether AaEA has an effect on immune cells. The active part of *A. argyi* was determined, but the metabolism of AaEA in vivo remains unknown. The metabolites’ more active in anti-inflammation and antiapoptosis need further investigation. Based on the present discovery, further study should be designed and conducted for the following aspects. Firstly, the effect and mechanism of AaEA administrating immune cells need to be studied in the next stage. Secondly, the chemical compositions of AaEA were complex, so systematic isolation and identification of the component from AaEA are necessary. Thirdly, the mechanism of compounds against ILI needs to be further clarified. The structure–activity relationship and structural optimization of the active compounds should be studied as well, which can provide a strategy to design lead compounds for anti-immunological immune liver injury. Last, what calls for special attention is the metabolism of AaEA in vivo and the activities of the metabolism. It is necessary to study not only the serum chemical parameters but also the pharmacokinetics parameters in the future.

In summary, this study clarified the phytochemical components of AaEA, which have various bioactivities, and mitigated inflammation and oxidative stress caused by ILI. The results demonstrated that pretreatment with AaEA dramatically inhibited the development of ILI in mice by regulating serum aminotransferases, cytokine production, hepatic inflammation, and cell apoptosis. Further study showed that AaEA via mediating Bax/Bcl-2 and TLR4/MyD88/NF-*κ*B signaling pathways suppressed inflammation and apoptosis in the process of ILI. The results provide a theoretical basis for *A. argyi* applied in diet therapy and functional health food production, which would be a great assistance for potential screening strategies for chronic liver diseases.

## Data Availability

All data were generated in-house, and no paper mill was used. All authors agree to be accountable for all aspects of work ensuring integrity and accuracy.

## Supplementary Materials

The following supporting information can be downloaded at: https://www.mdpi.com/article/10.3390/molecules27227883/s1, Supplementary Materials S1–S9: Chemical components from the ethyl a cetate extract of *Artemisia argyi* analyzed by LC-MS.

## Abbreviations

*Artemisia argyi* ethyl acetate extract, (AaEA); *Artemisia argyi* Lévl. et Vant., (*A. argyi*); alanine aminotransferase, (ALT); aspartate aminotransferase, (AST); Bcl-2-associated x, (Bax); B-cell lymphoma-2, (Bcl-2); Concanavalin A, (ConA); diammonium glycyrrhizinate, (DIG); glutathione peroxidase, (GSH-px); hematoxylin and eosin, (H&E); interferon-*γ*, (IFN-*γ*); inhibitor of NF-*κ*B, (I*κ*B); inhibitor of kappa B kinase, (IKK); immunological liver injury, (ILI); lipopolysaccharide, (LPS); liquid chromatograph-mass spectrometer, (LC-MS); high-, middle-, and low-dose of AaEA groups, (AaEA-H, AaEA-M, AaEA-L); malondialdehyde, (MDA); myeloid differentiation factor 88, (MyD88); one-way analysis of variance, (ANOVA); nuclear factor kappa beta, (NF-*κ*B); superoxide dismutase, (SOD); tumor necrosis factor-*α*, (TNF-*α*); TdT-mediated dUTP nick-end labeling, (TUNEL); Toll-like receptors, (TLRs).
