# Supplementary material for: Ethyl Acetate Extract from Artemisia argyi Prevents Liver Damage in ConA-Induced Immunological Liver Injury Mice via Bax/Bcl-2 and TLR4/MyD88/NF-κB Signaling Pathways
Source: Molecules. 2022 Nov 15;27(22):7883. doi: 10.3390/molecules27227883 (PMC9693258; doi:10.3390/molecules27227883)

Ethyl acetate extract from *Artemisia argyi* prevents liver damage in ConA-induced immunological liver injury mice via Bax/Bcl-2 and TLR4/MyD88/NF-*κ*B signaling pathways

Wen-qian Yang[a], Fei Shao[a], Jie-xin Wang[a], Tong Shen[a], Yu Zhao[a], Xue-yan Fu[a, b, c], Li-ming Zhang[a, b, c, *] and Hang-ying Li[a, b, c, *]

College of Pharmacy, Ningxia Medical University, Yinchuan, China.

Corresponding Author

E-mail: lihy17@lzu.edu.cn (Hangying Li).

Table of content **Page**

Chemical components from the ethyl a cetate extract of *Artemisia argyi* analyzed by LC-MS

SI 1. Identification of flavonoids from AaEA....................3

SI 2. Identification of lignans and coumarins from AaEA....................14

SI 3. Identification of alkaloids from AaEA....................17

SI 4. Identification of terpenoids from AaEA....................19

SI 5. Identification of phenolic acids from AaEA....................22

SI 6. Identification of others from AaEA....................28

SI 7. The primers used for Real-time PCR analysis....................30

SI 8. Schematic diagram of regulating Bax/Bcl-2 and TLR4/MyD88/NF-*κ*B pathways in the treatment of the immune liver injury....................31

SI 9. The original images of Western Blot....................32

SI 1. Identification of flavonoids from AaEA

Compounds	Formula	Molecular Weight (Da)	Ionization model
Pinocembrin (Dihydrochrysin)	$C_{15}H_{12}O_4$	256.07	$[M-H]^-$
3',4',7-Trihydroxyflavone	$C_{15}H_{10}O_5$	270.05	$[M+H]^+$
Apigenin	$C_{15}H_{10}O_5$	270.05	$[M+H]^+$
Galangin (3,5,7-Trihydroxyflavone)	$C_{15}H_{10}O_5$	270.05	$[M-H]^-$
Naringenin (5,7,4'-Trihydroxyflavanone)*	$C_{15}H_{10}O_5$	272.07	$[M-H]^-$
Naringenin chalcone*	$C_{15}H_{10}O_5$	272.07	$[M+H]^+$
Pinobanksin	$C_{15}H_{12}O_5$	272.07	$[M-H]^-$
Genkwanin (Apigenin 7-methyl ether)	$C_{16}H_{12}O_5$	284.07	$[M+H]^+$
Wogonin (5,7-Dihydroxy-8-Methoxyflavone)	$C_{16}H_{12}O_5$	284.07	$[M+H]^+$
Isoscutellarein	$C_{15}H_{10}O_6$	286.05	$[M+H]^+$
Scutellarein (5,6,7,4'-Tetrahydroxyflavone)	$C_{15}H_{10}O_6$	286.05	$[M+H]^+$
Luteolin	$C_{15}H_{10}O_6$	286.05	$[M+H]^+$
Kaempferol (3,5,7,4'-Tetrahydroxyflavone)	$C_{15}H_{10}O_6$	286.05	$[M-H]^-$
Okanin	$C_{15}H_{12}O_6$	288.06	$[M+H]^+$
2-hydroxynaringenin	$C_{15}H_{12}O_6$	288.06	$[M-H]^-$
Eriodictyol (5,7,3',4'-Tetrahydroxyflavanone)	$C_{15}H_{12}O_6$	288.06	$[M+H]^+$
3,4,2',4',6'-Pentahydroxychalcone	$C_{15}H_{12}O_6$	288.06	$[M-H]^-$
Catechin	$C_{15}H_{14}O_6$	290.08	$[M-H]^-$
Apigenin-7,4'-dimethyl ether	$C_{17}H_{14}O_5$	298.08	$[M+H]^+$
6-methyl luteolin	$C_{16}H_{12}O_6$	300.06	$[M+H]^+$
Rhamnocitrin (7-Methylkaempferol)	$C_{16}H_{12}O_6$	300.06	$[M+H]^+$
5,7,2'-Trhiyroxy-8-methoxyflavone	$C_{16}H_{12}O_6$	300.06	$[M-H]^-$

Compounds	Formula	Molecular Weight (Da)	Ionization model
Hispidulin (5,7,4'-Trihydroxy-6-methoxyflavone)	$C_{16}H_{12}O_6$	300.06	$[M+H]^+$
6,7,8-Tetrahydroxy-5-methoxyflavone	$C_{16}H_{12}O_6$	300.06	$[M+H]^+$
Ladanetin	$C_{16}H_{12}O_6$	300.06	$[M+H]^+$
Diosmetin (5,7,3'-Trihydroxy-4'-methoxyflavone)	$C_{16}H_{12}O_6$	300.06	$[M-H]^-$
Hypolaetin	$C_{15}H_{10}O_7$	302.04	$[M-H]^-$
Isoetin (5,7,2',4',5'-Pentahydroxyflavone)	$C_{15}H_{10}O_7$	302.04	$[M-H]^-$
6-HydroxyLuteolin	$C_{15}H_{10}O_7$	302.04	$[M-H]^-$
Quercetin	$C_{15}H_{10}O_7$	302.04	$[M+H]^+$
Robinetin	$C_{15}H_{10}O_7$	302.04	$[M+H]^+$
Tricetin (5,7,3',4',5'-Pentahydroxyflavone)	$C_{15}H_{10}O_7$	302.04	$[M-H]^-$
Dihydrokaempferide	$C_{16}H_{14}O_6$	302.08	$[M+H]^+$
Hesperetin	$C_{16}H_{14}O_6$	302.08	$[M-H]^-$
Homoeriodictyol	$C_{16}H_{14}O_6$	302.08	$[M+H]^+$
Dihydroquercetin(Taxifolin)	$C_{15}H_{12}O_7$	304.06	$[M-H]^-$
5,7,3',4',5'-Pentahydroxydihydroflavone	$C_{15}H_{12}O_7$	304.06	$[M-H]^-$
Isobavachalcone D	$C_{19}H_{20}O_4$	312.14	$[M+H]^+$
Dihydroxy-dimethoxyflavone	$C_{17}H_{14}O_6$	314.08	$[M+H]^+$
Pectolinarigenin	$C_{17}H_{14}O_6$	314.08	$[M+H]^+$
3’,4’-Dihydroxy-7,5’-dimethoxyflavone	$C_{17}H_{14}O_6$	314.08	$[M+H]^+$
3-O-Acetylpinobanksin	$C_{17}H_{14}O_6$	314.08	$[M-H]^-$
4',5-Dihydroxy-3',5'-dimethoxyflavone	$C_{17}H_{14}O_6$	314.08	$[M+H]^+$
5,2'-Dihydroxy-7,8-dimethoxyflavone	$C_{17}H_{14}O_6$	314.08	$[M+H]^+$
Cirsimaritin	$C_{17}H_{14}O_6$	314.29	$[M+H]^+$

Compounds	Formula	Molecular Weight (Da)	Ionization model
Azaleatin (5-O-Methylquercetin)	$C_{16}H_{12}O_7$	316.06	$[M+H]^+$
Tamarixetin (3,3',5,7-Tetrahydroxy-4'-methoxyflavone)	$C_{16}H_{12}O_7$	316.06	$[M+H]^+$
Isorhamnetin	$C_{16}H_{12}O_7$	316.06	$[M-H]^-$
Rhamnetin	$C_{16}H_{12}O_7$	316.06	$[M-H]^-$
Nepetin (5,7,3',4'-Tetrahydroxy-6-methoxyflavone)	$C_{16}H_{12}O_7$	316.06	$[M+H]^+$
3-O-Methylquercetin	$C_{16}H_{12}O_7$	316.06	$[M-H]^-$
Isobavachin	$C_{20}H_{20}O_4$	324.37	$[M+H]^+$
Eucalyptin (5-Hydroxy-7,4'-dimethoxy-6,8-dimethylflavone)	$C_{19}H_{18}O_5$	326.12	$[M-H]^-$
Salvigenin	$C_{18}H_{16}O_6$	328.09	$[M+H]^+$
6-Hydroxy-5,7,4'-trimethoxyflavone	$C_{18}H_{16}O_6$	328.10	$[M+H]^+$
Rehderianin I	$C_{17}H_{14}O_7$	330.07	$[M+H]^+$
Ombuin (4',7-Dimethoxy-3,3',5-Trihydroxyflavone)	$C_{17}H_{14}O_7$	330.07	$[M+H]^+$
tricin (5,7,4'-Trihydroxy-3',5'-dimethoxyflavone)	$C_{17}H_{14}O_7$	330.07	$[M+H]^+$
Cirsiliol (3',4',5-Trihydroxy-6,7-Dimethoxyflavone)	$C_{17}H_{14}O_7$	330.07	$[M+H]^+$
4',5,7-Trihydroxy-3',6-dimethoxyflavone (Jaceosidin)	$C_{17}H_{14}O_7$	330.07	$[M+H]^+$
7-Hydroxy-2',5,8-Trimethoxyflavanone	$C_{18}H_{18}O_6$	330.11	$[M+H]^+$
Patuletin (Quercetagetin-6-methyl ether)	$C_{16}H_{12}O_8$	332.05	$[M-H]^-$
7,8-Dihydroxy-5,6,4'-trimethoxyflavone	$C_{18}H_{16}O_7$	344.09	$[M+H]^+$

Compounds	Formula	Molecular Weight (Da)	Ionization model
Tenaxin I	$C_{18}H_{16}O_7$	344.09	$[M+H]^+$
Pachypodol (5,4'-dihydroxy-3,7,3'-trimethoxyflavone)	$C_{18}H_{16}O_7$	344.09	$[M+H]^+$
Cirsilineol (4',5-Dihydroxy-3',6,7-trimethoxyflavone)	$C_{18}H_{16}O_7$	344.09	$[M-H]^-$
Ayanin (3',5-Dihydroxy-3,4',7-Trimethoxyflavone)	$C_{18}H_{16}O_7$	344.09	$[M+H]^+$
Eupatorin	$C_{18}H_{16}O_7$	344.09	$[M+H]^+$
Penduletin (5,4'-Dihydroxy-3,6,7-trimethoxyflavone)	$C_{18}H_{16}O_7$	344.09	$[M+H]^+$
5,6,7,4'-Tetramethoxyflavanone	$C_{19}H_{20}O_6$	344.13	$[M+H]^+$
Eupatilin	$C_{18}H_{16}O_7$	344.32	$[M+H]^+$
Quercetagetin-3,4'-Dimethyl Ether	$C_{17}H_{14}O_8$	346.07	$[M+H]^+$
Syringetin	$C_{17}H_{14}O_8$	346.07	$[M-H]^-$
5,7,4',5'-Tetrahydro-3',6-dimethoxyflavone	$C_{17}H_{14}O_8$	346.07	$[M+H]^+$
3,3',5,7-Tetrahydroxy-4',6-Dimethoxyflavone; (Laciniatin)	$C_{17}H_{14}O_8$	346.07	$[M+H]^+$
7-Hydroxy-3,5,6,8-tetramethoxyflavone	$C_{19}H_{18}O_7$	358.11	$[M+H]^+$
5-Hydroxy-6,7,3',4'-tetramethoxyflavone	$C_{19}H_{18}O_7$	358.11	$[M+H]^+$
Eupatorin-5-methylether (3'-hydroxy-5,6,7,4'-tetramethoxyflavone)	$C_{19}H_{18}O_7$	358.11	$[M+H]^+$
Centaureidin (5,7,3'-Trihydroxy-3,6,4'-Trimethoxyflavone)	$C_{18}H_{16}O_8$	360.08	$[M+H]^+$
Chrysosplenol D	$C_{18}H_{16}O_8$	360.08	$[M+H]^+$

Compounds	Formula	Molecular Weight (Da)	Ionization model
Thymonin	$C_{18}H_{16}O_8$	360.09	$[M+H]^+$
Arcapillin (2',4',5-trihydroxy-5',6,7-trimethoxyflavone)	$C_{18}H_{16}O_8$	360.09	$[M+H]^+$
4',5,7-Trihydroxy-3',3,6-Trimethoxyflavone (Jaceidin)	$C_{18}H_{16}O_8$	360.09	$[M+H]^+$
Acerosin	$C_{18}H_{16}O_8$	360.09	$[M-H]^-$
Sudachitin	$C_{18}H_{16}O_8$	360.09	$[M-H]^-$
Tangeretin (4',5,6,7,8-Pentamethoxyflavone)	$C_{20}H_{20}O_7$	372.12	$[M+H]^+$
Chrysosplenetin (Quercetagetin-3,6,7,3'-tetramethyl ether)	$C_{19}H_{18}O_8$	374.10	$[M+H]^+$
Casticin	$C_{19}H_{18}O_8$	374.10	$[M+H]^+$
5,4′-Dihydroxy-3,6,7,3′-tetramethoxyflavone	$C_{19}H_{18}O_8$	374.10	$[M+H]^+$
Skullcapflavone II	$C_{19}H_{18}O_8$	374.10	$[M+H]^+$
5,7-Dihydroxy-6,3',4',5'-tetramethoxyflavone (Arteanoflavone)	$C_{19}H_{18}O_8$	374.10	$[M+H]^+$
5-Demethylnobiletin	$C_{20}H_{20}O_8$	388.12	$[M+H]^+$
Artemetin (5-Hydroxy-3,3',4',6,7-Pentamethoxyflavone)	$C_{20}H_{20}O_8$	388.12	$[M+H]^+$
Nobiletin (5,6,7,8,3',4'-Hexamethoxyflavone)	$C_{21}H_{22}O_8$	402.13	$[M+H]^+$
2'-Hydroxy-3,4,5,3',4',6'-hexamethoxychalcone	$C_{21}H_{24}O_8$	404.15	$[M+H]^+$
Apigenin-6-C-glucoside (Isovitexin)	$C_{21}H_{20}O_{10}$	432.11	$[M-H]^-$
Isovitexin	$C_{21}H_{20}O_{10}$	432.11	$[M+H]^+$
Apigenin-5-O-glucoside	$C_{21}H_{20}O_{10}$	432.11	$[M+H]^+$
Galangin-7-O-glucoside	$C_{21}H_{20}O_{10}$	432.11	$[M+H]^+$

Compounds	Formula	Molecular Weight (Da)	Ionization model
3,5,6,7,8,3',4'-Heptamethoxyflavone	$C_{22}H_{24}O_9$	432.14	$[M+H]^+$
Avicularin(Quercetin-3-O-α-L-arabinofuranoside)	$C_{20}H_{18}O_{11}$	434.09	[M+H]+
Quercetin-3-O-arabinoside (Guaijaverin)	$C_{20}H_{18}O_{11}$	434.09	$[M-H]^-$
Quercetin-3-O-xyloside (Reynoutrin)	$C_{20}H_{18}O_{11}$	434.09	$[M+H]^+$
Isohemiphloin	$C_{21}H_{22}O_{10}$	434.12	$[M-H]^-$
Butin-7-O-glucoside	$C_{21}H_{22}O_{10}$	434.12	$[M-H]^-$
Naringenin-4'-O-glucoside	$C_{21}H_{22}O_{10}$	434.12	$[M-H]^-$
Naringenin-7-O-glucoside (Prunin)	$C_{21}H_{22}O_{10}$	434.12	$[M-H]^-$
choerospondin	$C_{21}H_{22}O_{10}$	434.12	$[M-H]^-$
Phloretin-4'-O-glucoside (Trilobatin)	$C_{21}H_{24}O_{10}$	436.14	$[M-H]^-$
Leachianone A	$C_{26}H_{30}O_6$	438.20	$[M+H]^+$
Apigenin-7-O-glucuronide	$C_{21}H_{18}O_{11}$	446.09	$[M-H]^-$
Baicalin	$C_{21}H_{18}O_{11}$	446.09	$[M+H]^+$
Isorhamnetin-3-O-arabinoside	$C_{21}H_{20}O_{11}$	448.10	$[M+H]^+$
Kaempferol-4'-O-glucoside	$C_{21}H_{20}O_{11}$	448.10	$[M+H]^+$
Luteolin-8-C-glucoside (orientin)	$C_{21}H_{20}O_{11}$	448.10	$[M+H]^+$
Kaempferol-7-O-glucoside	$C_{21}H_{20}O_{11}$	448.10	$[M-H]^-$
Luteolin-6-C-glucoside (Isoorientin)	$C_{21}H_{20}O_{11}$	448.10	$[M-H]^-$
Carthamone	$C_{21}H_{20}O_{11}$	448.10	$[M-H]^-$
Luteolin-7-O-glucoside (Cynaroside)	$C_{21}H_{20}O_{11}$	448.10	$[M+H]^+$
Kaempferol-3-O-galactoside (Trifolin)	$C_{21}H_{20}O_{11}$	448.10	$[M-H]^-$
Kaempferol-3-O-glucoside (Astragalin)	$C_{21}H_{20}O_{11}$	448.10	$[M+H]^+$
Luteolin-4'-O-glucoside	$C_{21}H_{20}O_{11}$	448.10	$[M+H]^+$

Compounds	Formula	Molecular Weight (Da)	Ionization model
Luteolin-3'-O-glucoside	$C_{21}H_{20}O_{11}$	448.10	$[M+H]^+$
6-C-Glucosyl-2-Hydroxynaringenin	$C_{21}H_{22}O_{11}$	450.12	$[M-H]^-$
eriodictyol 7-O-β-D-glucopyranoside	$C_{21}H_{22}O_{11}$	450.12	$[M+H]^+$
Okanin-4'-O-glucoside(Marein)	$C_{21}H_{22}O_{11}$	450.12	$[M+H]^+$
Aromadendrin-7-O-glucoside	$C_{21}H_{22}O_{11}$	450.12	$[M-H]^-$
Dihydrokaempferol-3-O-glucoside	$C_{21}H_{22}O_{11}$	450.12	$[M+H]^+$
Eriodictyol-3'-O-glucoside	$C_{21}H_{22}O_{11}$	450.12	$[M-H]^-$
Dihydrokaempferol-7-O-glucoside	$C_{21}H_{22}O_{11}$	450.12	$[M-H]^-$
Eriodictyol-7-O-glucoside	$C_{21}H_{22}O_{11}$	450.12	$[M+H]^+$
3,4,2',4',6'-Pentahydroxychalcone-4'-O-glucoside	$C_{21}H_{22}O_{11}$	450.12	$[M-H]^-$
3',5,5',7-Tetrahydroxyflavanone-7-O-glucoside	$C_{21}H_{22}O_{11}$	450.12	$[M+H]^+$
Kaempferol-3-O-glucuronide	$C_{21}H_{18}O_{12}$	462.08	$[M-H]^-$
Scutellarin	$C_{21}H_{18}O_{12}$	462.08	$[M+H]^+$
Luteolin-7-O-glucuronide	$C_{21}H_{18}O_{12}$	462.08	$[M+H]^+$
Diosmetin-7-O-Beta-D-Glucopyranoside	$C_{22}H_{22}O_{11}$	462.12	$[M+H]^+$
Yuanhuanin	$C_{22}H_{22}O_{11}$	462.12	$[M+H]^+$
Diosmetin-7-O-galactoside	$C_{22}H_{22}O_{11}$	462.12	$[M+H]^+$
Chrysoeriol-7-O-glucoside	$C_{22}H_{22}O_{11}$	462.12	$[M-H]^-$
6-C-MethylKaempferol-3-glucoside	$C_{22}H_{22}O_{11}$	462.12	$[M+H]^+$
Quercetin-7-O-glucoside*	$C_{21}H_{20}O_{12}$	464.10	$[M-H]^-$
Quercetin-4'-O-glucoside (Spiraeoside)*	$C_{21}H_{20}O_{12}$	464.10	$[M-H]^-$
6-HydroxyLuteolin 5-glucoside	$C_{21}H_{20}O_{12}$	464.10	$[M-H]^-$
6-Hydroxykaempferol-7-O-glucoside	$C_{21}H_{20}O_{12}$	464.10	$[M+H]^+$
Quercetin-3-O-glucoside (Isoquercitrin)*	$C_{21}H_{20}O_{12}$	464.10	$[M-H]^-$

Compounds	Formula	Molecular Weight (Da)	Ionization model
Isohyperoside	$C_{21}H_{20}O_{12}$	464.10	$[M+H]^+$
Quercetin-5-O-β-D-glucoside*	$C_{21}H_{20}O_{12}$	464.10	$[M+H]^+$
Hesperetin-7-O-glucoside	$C_{22}H_{24}O_{11}$	464.13	$[M+H]^+$
Hesperetin-5-O-glucoside	$C_{22}H_{24}O_{11}$	464.13	$[M-H]^-$
Taxifolin-3'-O-glucoside	$C_{21}H_{22}O_{12}$	466.11	$[M+H]^+$
Diosmetin-7-O-glucuronide	$C_{22}H_{20}O_{12}$	476.10	$[M+H]^+$
Quercetin-3-O-glucuronide	$C_{21}H_{18}O_{13}$	478.08	$[M+H]^+$
6-Methoxykaempferol-3-O-glucoside	$C_{22}H_{22}O_{12}$	478.11	$[M+H]^+$
Isorhamnetin-7-O-glucoside (Brassicin)	$C_{22}H_{22}O_{12}$	478.11	$[M+H]^+$
Rhamnetin-3-O-Glucoside	$C_{22}H_{22}O_{12}$	478.11	$[M+H]^+$
Isorhamnetin-3-O-Glucoside	$C_{22}H_{22}O_{12}$	478.11	$[M+H]^+$
Nepetin-7-O-glucoside	$C_{22}H_{22}O_{12}$	478.11	$[M+H]^+$
Persicoside	$C_{23}H_{26}O_{11}$	478.15	$[M-H]^-$
Myricetin-3-O-galactoside	$C_{21}H_{20}O_{13}$	480.09	$[M+H]^+$
Myricetin-3-O-glucoside	$C_{21}H_{20}O_{13}$	480.09	$[M-H]^-$
Silymarin	$C_{25}H_{22}O_{10}$	482.44	$[M-H]^-$
Kaempferol-3-O-(6"-acetyl)glucoside	$C_{23}H_{22}O_{12}$	490.11	$[M-H]^-$
tricin-7-O-Glucoside	$C_{23}H_{24}O_{12}$	492.13	$[M+H]^+$
Jaceosidin-7-O-Glucoside	$C_{23}H_{24}O_{12}$	492.13	$[M+H]^+$
Laricitrin-3-O-glucoside	$C_{22}H_{22}O_{13}$	494.11	$[M+H]^+$
Patuletin-3-O-glucoside	$C_{22}H_{22}O_{13}$	494.11	$[M+H]^+$
Mearnsetin-3-O-glucoside	$C_{22}H_{22}O_{13}$	494.11	$[M-H]^-$
Chrysoeriol-7-O-(6"-acetyl)glucoside	$C_{24}H_{24}O_{12}$	504.13	$[M-H]^-$
Eupatilin-7-O-glucoside	$C_{24}H_{26}O_{12}$	506.14	$[M+H]^+$

Compounds	Formula	Molecular Weight (Da)	Ionization model
Limocitrin-3-O-galactoside	$C_{23}H_{24}O_{13}$	508.12	$[M+H]^+$
Limocitrin-3-O-glucoside	$C_{23}H_{24}O_{13}$	508.12	$[M+H]^+$
5,6,3′,4′-Tetrahydroxy-3,7-dimethoxyflavone-6-O-glucoside	$C_{23}H_{24}O_{13}$	508.12	$[M+H]^+$
Syringetin-7-O-glucoside	$C_{23}H_{24}O_{13}$	508.12	$[M+H]^+$
Epimedoside C	$C_{26}H_{28}O_{11}$	516.16	$[M+H]^+$
Kaempferol-3-O-(6"-malonyl)glucoside	$C_{24}H_{22}O_{14}$	534.10	$[M+H]^+$
Kaempferol-3-O-(6"-malonyl)galactoside	$C_{24}H_{22}O_{14}$	534.10	$[M+H]^+$
Apigenin-6,8-di-C-arabinoside	$C_{25}H_{26}O_{13}$	534.14	$[M+H]^+$
Apigenin-6-C-xyloside-8-C-arabinoside	$C_{25}H_{26}O_{13}$	534.14	$[M+H]^+$
Quercetin-7-O-(6"-malonyl)glucoside	$C_{24}H_{22}O_{15}$	550.10	$[M+H]^+$
Isorhamnetin-3-O-(6"-malonylglucoside)	$C_{25}H_{24}O_{15}$	564.09	$[M+H]^+$
Apigenin-6-C-arabinoside-8-C-glucoside (Isoschaftoside)*	$C_{26}H_{28}O_{14}$	564.12	$[M+H]^+$
Apigenin-6-C-(2"-glucosyl)arabinoside	$C_{26}H_{28}O_{14}$	564.15	$[M+H]^+$
Hispidulin-8-C-(2"-O-xylosyl)xyloside	$C_{26}H_{28}O_{14}$	564.15	$[M+H]^+$
Apigenin-6-C-(2"-xylosyl)glucoside	$C_{26}H_{28}O_{14}$	564.15	$[M+H]^+$
Isovitexin-2"xyloside	$C_{26}H_{28}O_{14}$	564.15	$[M+H]^+$
Phloretin-4'-O-(6"-cinnamoyl)glucoside	$C_{30}H_{30}O_{11}$	566.18	$[M-H]^-$
Apigenin-7-O-(6"-p-Coumaryl)glucoside	$C_{30}H_{26}O_{12}$	578.14	$[M+H]^+$
Apigenin-7-O-rutinoside (Isorhoifolin)	$C_{27}H_{30}O_{14}$	578.16	$[M+H]^+$
Rhoifolin	$C_{27}H_{30}O_{14}$	578.52	$[M+H]^+$
Kaempferol-3-O-sambubioside	$C_{26}H_{28}O_{15}$	580.14	$[M+H]^+$
Naringenin-7-O-Rutinoside(Narirutin)*	$C_{27}H_{32}O_{14}$	580.18	$[M-H]^-$

Compounds	Formula	Molecular Weight (Da)	Ionization model
Naringenin-7-O-Neohesperidoside(Naringin)*	$C_{27}H_{32}O_{14}$	580.18	$[M-H]^-$
Chrysoeriol-6-C-rhamnoside-7-O-rhamnoside	$C_{28}H_{32}O_{14}$	592.18	$[M+H]^+$
Robinson-7-O-Neohesperidin	$C_{28}H_{32}O_{14}$	592.18	$[M+H]^+$
Acacetin-7-O-rutinoside (Linarin)	$C_{28}H_{32}O_{14}$	592.18	$[M+H]^+$
Luteolin-7-O-(6"-caffeoyl)rhamnoside	$C_{30}H_{26}O_{13}$	594.14	$[M-H]^-$
Kaempferol-3-O-(6"-p-Coumaroyl)glucoside (Tiliroside)	$C_{30}H_{26}O_{13}$	594.14	$[M+H]^+$
Luteolin-7-O-neohesperidoside (lonicerin)	$C_{27}H_{30}O_{15}$	594.16	$[M+H]^+$
Luteolin-7-O-rutinoside	$C_{27}H_{30}O_{15}$	594.16	$[M+H]^+$
Kaempferol-3-O-neohesperidoside	$C_{27}H_{30}O_{15}$	594.16	$[M+H]^+$
Kaempferol-3-O-rutinoside(Nicotiflorin)	$C_{27}H_{30}O_{15}$	594.16	$[M+H]^+$
Apigenin-6,8-di-C-glucoside (Vicenin-2)	$C_{27}H_{30}O_{15}$	594.16	$[M+H]^+$
vitexin-2"-O-glucoside	$C_{27}H_{30}O_{15}$	594.16	$[M+H]^+$
Kaempferol-3-O-glucoside-7-O-rhamnoside	$C_{27}H_{30}O_{15}$	594.16	$[M+H]^+$
Isorhamnetin-3-O-arabinoside-7-O-rhamnoside	$C_{27}H_{30}O_{15}$	594.16	$[M+H]^+$
IsoSaponarin(Isovitexin-4'-O-glucoside)	$C_{27}H_{30}O_{15}$	594.16	$[M+H]^+$
Didymin (Isosakuranetin-7-O-rutinoside)	$C_{28}H_{34}O_{14}$	594.20	$[M-H]^-$
Kaempferol-3-O-glucorhamnoside	$C_{27}H_{30}O_{15}$	594.52	$[M+H]^+$
Quercetin-3-O-apiosyl(1→2)galactoside	$C_{26}H_{28}O_{16}$	596.14	$[M+H]^+$
Quercetin-3-O-sambubioside	$C_{26}H_{28}O_{16}$	596.14	$[M+H]^+$
Eriodictyol-7-O-Rutinoside (Eriocitrin)	$C_{27}H_{32}O_{15}$	596.17	$[M-H]^-$
Diosmetin-7-O-rutinoside (Diosmin)	$C_{28}H_{32}O_{15}$	608.17	$[M+H]^+$
Chrysoeriol-7-O-rutinoside	$C_{28}H_{32}O_{15}$	608.17	$[M-H]^-$
Diosmetin-7-O-Neohesperidoside (Neodiosmin)	$C_{28}H_{32}O_{15}$	608.17	$[M+H]^+$

Compounds	Formula	Molecular Weight (Da)	Ionization model
Hispidulin-7-O-(6"-O-p-Coumaroyl)Glucoside	$C_{31}H_{28}O_{13}$	608.18	$[M+H]^+$
Quercetin-3-O-glucoside-7-O-rhamnoside	$C_{27}H_{30}O_{16}$	610.15	$[M+H]^+$
Quercetin-3-O-robinobioside	$C_{27}H_{30}O_{16}$	610.15	$[M-H]^-$
Quercetin-3-O-rutinoside (Rutin)	$C_{27}H_{30}O_{16}$	610.15	$[M-H]^-$
Luteolin-7,3'-di-O-glucoside	$C_{27}H_{30}O_{16}$	610.15	$[M+H]^+$
Quercetin-3-O-neohesperidoside	$C_{27}H_{30}O_{16}$	610.15	$[M+H]^+$
Luteolin-7-O-gentiobioside	$C_{27}H_{30}O_{16}$	610.15	$[M+H]^+$
Tamarixetin-3-O-rutinoside	$C_{28}H_{32}O_{16}$	624.17	$[M-H]^-$
Chrysoeriol-7-O-gentiobioside	$C_{28}H_{32}O_{16}$	624.17	$[M+H]^+$
Isorhamnetin-3-O-neohespeidoside	$C_{28}H_{32}O_{16}$	624.17	$[M+H]^+$
Quercetin-5,4′-di-O-glucoside	$C_{27}H_{30}O_{17}$	626.15	$[M+H]^+$
6-Hydroxykaempferol 3,6-Diglucoside	$C_{27}H_{30}O_{17}$	626.15	$[M+H]^+$
Meratin	$C_{27}H_{30}O_{17}$	626.15	$[M+H]^+$
Quercetin-3-O-(2"-O-glucosyl)glucuronide	$C_{27}H_{28}O_{18}$	640.13	$[M+H]^+$
Patuletin-3-O-rutinoside	$C_{28}H_{32}O_{17}$	640.17	$[M+H]^+$
Tricin-5,7-O-diglucoside	$C_{29}H_{34}O_{17}$	654.18	$[M+H]^+$
Syringetin-3-O-rutinoside	$C_{29}H_{34}O_{17}$	654.18	$[M+H]^+$
Tricin-4'-O-glucoside-7-O-glucoside	$C_{29}H_{34}O_{17}$	654.18	$[M+H]^+$

SI 2. Identification of lignans and coumarins from AaEA

Compounds	Formula	Molecular Weight (Da)	Ionization model
Coumarin	$C_9H_6O_2$	146.04	$[M+H]^+$
6-MethylCoumarin	$C_{10}H_8O_2$	160.05	$[M+H]^+$
7-Hydroxycoumarin	$C_9H_6O_3$	162.03	$[M-H]^-$
7-Methoxycoumarin	$C_{10}H_8O_3$	176.05	$[M+H]^+$
6-Hydroxy-4-methylcoumarin	$C_{10}H_8O_3$	176.05	$[M-H]^-$
Esculetin	$C_9H_6O_4$	178.03	$[M-H]^-$
Daphnetin	$C_9H_6O_4$	178.14	$[M+H]^+$
Psoralen	$C_{11}H_6O_3$	186.03	$[M+H]^+$
Scopoletin (7-Hydroxy-6-methoxycoumarin)	$C_{10}H_8O_4$	192.04	$[M+H]^+$
Isoscopoletin (6-Hydroxy-7-Methoxycoumarin)	$C_{10}H_8O_4$	192.04	$[M+H]^+$
Scoparone	$C_{11}H_{10}O_4$	206.19	$[M+H]^+$
Fraxetin (7,8-Dihydroxy-6-methoxycoumarin)	$C_{10}H_8O_5$	208.04	$[M+H]^+$
Umckalin (7-hydroxy-5,6-dimethoxycoumarin)	$C_{11}H_{10}O_5$	222.05	$[M+H]^+$
isofraxidin	$C_{11}H_{10}O_5$	222.05	$[M+H]^+$
Fraxidin (8-Hydroxy-6,7-dimethoxycoumarin)	$C_{11}H_{10}O_5$	222.05	$[M+H]^+$
5,6,7-Trimethoxycoumarin	$C_{12}H_{12}O_5$	236.07	$[M+H]^+$
5,7,8-trimethoxycoumarin	$C_{12}H_{12}O_5$	236.07	$[M+H]^+$
Dimethylfraxetin	$C_{12}H_{12}O_5$	236.22	$[M+H]^+$
Demethylwedelolactone	$C_{15}H_8O_7$	300.03	$[M-H]^-$
Wedelolactone	$C_{16}H_{10}O_7$	314.25	$[M-H]^-$

Compounds	Formula	Molecular Weight (Da)	Ionization model
8-Geranyloxypsoralen	$C_{21}H_{22}O_4$	338.40	$[M+H]^+$
Daphnin	$C_{15}H_{16}O_9$	340.08	$[M-H]^-$
Esculetin-7-O-glucoside	$C_{15}H_{16}O_9$	340.08	$[M-H]^-$
Esculin (6,7-DihydroxyCoumarin-6-glucoside)	$C_{15}H_{16}O_9$	340.08	$[M-H]^-$
Turgeniifolin B	$C_{19}H_{22}O_6$	346.14	$[M+H]^+$
Sesamin	$C_{20}H_{18}O_6$	354.11	$[M+H]^+$
Pinoresinol	$C_{20}H_{22}O_6$	358.14	$[M-H]^-$
Epipinoresinol	$C_{20}H_{22}O_6$	358.14	$[M-H]^-$
scopoletin-7-O-glucuronide	$C_{16}H_{16}O_{10}$	368.07	$[M+H]^+$
6,7-Dihydroxycoumarin-7-O-(6'-acetyl)glucoside	$C_{16}H_{16}O_{10}$	368.07	$[M-H]^-$
Fraxetin-8-O-glucoside (Fraxin)	$C_{16}H_{18}O_{10}$	370.09	$[M-H]^-$
(8'R,7'S)-(-)-8-Hydroxy-α-conidendrin	$C_{20}H_{20}O_7$	372.12	$[M-H]^-$
Isofraxidin-7-O-glucoside	$C_{17}H_{20}O_{10}$	384.11	$[M+H]^+$
Fraxidin-8-O-glucoside	$C_{17}H_{20}O_{10}$	384.11	$[M+H]^+$
syringaresinol	$C_{22}H_{26}O_8$	418.16	$[M-H]^-$
Secoisolariciresinol-9'-O-xyloside	$C_{25}H_{34}O_{10}$	494.22	$[M-H]^-$
Pinoresinol-4-O-glucoside	$C_{26}H_{32}O_{11}$	520.19	$[M-H]^-$
lariciresinol-4'-O-glucoside	$C_{26}H_{34}O_{11}$	522.21	$[M-H]^-$
Secoisolariciresinol 4-O-glucoside	$C_{26}H_{36}O_{11}$	524.23	$[M-H]^-$
Sesamolinol-glucoside	$C_{26}H_{30}O_{12}$	534.17	$[M-H]^-$
1-Hydroxypinoresinol-4'-O-Glucoside	$C_{26}H_{32}O_{12}$	536.19	$[M-H]^-$
Nortrachelogenin-4-O-glucoside	$C_{26}H_{32}O_{12}$	536.19	$[M-H]^-$

Compounds	Formula	Molecular Weight (Da)	Ionization model
1-Hydroxypinoresinol-1-O-Glucoside	$C_{26}H_{32}O_{12}$	536.19	$[M-H]^-$
5'-Methoxymatairesinoside	$C_{27}H_{34}O_{12}$	550.20	$[M-H]^-$
Eucommin A	$C_{27}H_{34}O_{12}$	550.21	$[M-H]^-$

SI 3. Identification of alkaloids from AaEA

Compounds	Formula	Molecular Weight (Da)	Ionization model
Triethylamine	$C_6H_{15}N$	101.12	$[M+H]^+$
Betaine	$C_5H_{11}NO_2$	117.08	$[M+H]^+$
N-Benzylmethylene isomethylamine	C_8H_9N	119.07	$[M+H]^+$
Phenethylamine	$C_8H_{11}N$	121.09	$[M+H]^+$
6-Deoxyfagomine	$C_6H_{13}NO_2$	131.10	$[M+H]^+$
N-benzylformamide	C_8H_9NO	135.07	$[M+H]^+$
Trigonelline	$C_7H_7NO_2$	137.05	$[M+H]^+$
Methyl L-pyroglutamate	$C_6H_9NO_3$	143.14	$[M+H]^+$
2-Acetyl-3-ethylpyrazine	$C_8H_{10}N_2O$	150.08	$[M+H]^+$
1,4-Dihydro-1-Methyl-4-oxo-3-pyridinecarboxamide	$C_7H_8N_2O_2$	152.06	$[M-H]^-$
Gentianine	$C_{10}H_9NO_2$	175.06	$[M+H]^+$
4-hydroxy-5-(2-oxo-l-pyrrolidinyl)-benzoic Acid	$C_9H_{11}NO_3$	181.07	$[M+H]^+$
Gentianamine	$C_{11}H_{11}NO_3$	205.07	$[M+H]^+$
epiguaipyridine	$C_{15}H_{21}N$	215.17	$[M+H]^+$
Patchoulipyridine	$C_{15}H_{21}N$	215.17	$[M+H]^+$
Caffeoylcholine	$C_{14}H_{20}NO_4^+$	266.12	[M]+
Laurocapram	$C_{18}H_{35}NO$	281.48	$[M+H]^+$
p-Coumaroyltyramine	$C_{17}H_{17}NO_3$	283.12	$[M+H]^+$
N-Oleoylethanolamine	$C_{20}H_{39}NO_2$	325.30	$[M+H]^+$
Cocamidopropyl betaine	$C_{19}H_{38}N_2O_3$	342.29	$[M+H]^+$

SI 4. Identification of terpenoids from AaEA

Compounds	Formula	Molecular Weight (Da)	Ionization model
Arteannuin A	$C_{13}H_{18}O_2$	206.13	$[M+H]^+$
Dehydrocostuslactone	$C_{15}H_{18}O_2$	230.13	$[M+H]^+$
Isoalantolactone	$C_{15}H_{20}O_2$	232.15	$[M+H]^+$
Arteannuin J	$C_{15}H_{22}O_2$	234.16	$[M-H]^-$
costic acid	$C_{15}H_{22}O_2$	234.16	$[M+H]^+$
pterodontic acid	$C_{15}H_{22}O_2$	234.16	$[M+H]^+$
10α-Hydroxycadin-4-en-15-al	$C_{15}H_{24}O_2$	236.18	$[M+H]^+$
Dihydroartemisinic acid	$C_{15}H_{24}O_2$	236.35	$[M+H]^+$
Lettucenin A	$C_{15}H_{12}O_3$	240.08	$[M+H]^+$
Lettucenin B	$C_{15}H_{14}O_3$	242.09	$[M+H]^+$
Dehydroleucodine	$C_{15}H_{16}O_3$	244.11	$[M+H]^+$
Yomogin	$C_{15}H_{16}O_3$	244.11	$[M+H]^+$
Inunal	$C_{15}H_{18}O_3$	246.13	$[M+H]^+$
Leucodin	$C_{15}H_{18}O_3$	246.13	$[M+H]^+$
Santonin	$C_{15}H_{18}O_3$	246.13	$[M+H]^+$
Cichoralexin	$C_{15}H_{20}O_3$	248.14	$[M+H]^+$
Britanilide	$C_{15}H_{20}O_3$	248.14	$[M+H]^+$
Parthenolide	$C_{15}H_{20}O_3$	248.14	$[M+H]^+$
Reynosin	$C_{15}H_{20}O_3$	248.14	[M−H]−
Arteannuin B	$C_{15}H_{20}O_3$	248.32	$[M+H]^+$
Arteannuin N	$C_{15}H_{22}O_3$	250.16	$[M+H]^+$

Compounds	Formula	Molecular Weight (Da)	Ionization model
5α-Hydroxycostic acid	$C_{15}H_{22}O_3$	250.16	$[M-H]^-$
Arteannuin K	$C_{15}H_{22}O_3$	250.16	$[M+H]^+$
Dihydro-epi-arteannuin B	$C_{15}H_{22}O_3$	250.16	$[M-H]^-$
Arteannuin L	$C_{15}H_{22}O_3$	250.16	$[M+H]^+$
Ilicic acid	$C_{15}H_{24}O_3$	252.17	$[M+H]^+$
8-Deoxylactucin	$C_{15}H_{16}O_4$	260.11	$[M+H]^+$
Austricin	$C_{15}H_{18}O_4$	262.12	$[M+H]^+$
Jacquinelin	$C_{15}H_{18}O_4$	262.12	$[M+H]^+$
Armexifolin	$C_{15}H_{18}O_4$	262.12	$[M-H]^-$
Ridentin	$C_{15}H_{20}O_4$	264.14	$[M+H]^+$
Artecalin	$C_{15}H_{20}O_4$	264.14	$[M+H]^+$
Arsanin	$C_{15}H_{22}O_4$	266.15	$[M-H]^-$
Artemin	$C_{15}H_{22}O_4$	266.15	$[M+H]^+$
Tanaphillin	$C_{15}H_{18}O_5$	278.12	$[M+H]^+$
3-Hydroxy-3,7,11-trimethyldodeca-1,6E,10-trien-9-yl propionate	$C_{17}H_{28}O_3$	280.20	$[M+H]^+$
3-Methoxytanapartholide	$C_{16}H_{20}O_5$	292.13	$[M+H]^+$
3-Hydroxy-3,7,11-trimethyldodeca-1,6E,10-trien-9-yl isobutyrate	$C_{18}H_{30}O_3$	294.22	$[M+H]^+$
Matricarin	$C_{17}H_{20}O_5$	304.13	$[M+H]^+$
Oxobritannilactone	$C_{17}H_{22}O_5$	306.15	$[M+H]^+$
Arvestolide B	$C_{17}H_{22}O_5$	306.15	$[M+H]^+$
Argyinolide B	$C_{17}H_{20}O_6$	320.13	$[M+H]^+$

Compounds	Formula	Molecular Weight (Da)	Ionization model
Moxartenolide	$C_{20}H_{22}O_5$	342.15	$[M+H]^+$
Argyinolide J	$C_{20}H_{28}O_5$	348.19	$[M+H]^+$
Balsamiferine E	$C_{20}H_{32}O_5$	352.23	$[M+H]^+$
Citroside A	$C_{19}H_{30}O_8$	386.19	$[M+H]^+$
Blinin	$C_{22}H_{32}O_6$	392.49	$[M-H]^-$
Ixerisoside D	$C_{21}H_{28}O_8$	408.18	$[M-H]^-$
Sonchuside G	$C_{21}H_{34}O_8$	414.23	$[M+H]^+$
pterodontoside E	$C_{21}H_{38}O_8$	418.26	$[M+H]^+$
2,3-dihydroxy-12-ursen-28-oic acid	$C_{30}H_{48}O_4$	472.36	$[M-H]^-$
Pomolic acid	$C_{30}H_{48}O_4$	472.36	$[M-H]^-$
Rubusic acid (3β,7α-Dihydroxyolean-12-en-28-oic acid)	$C_{30}H_{48}O_4$	472.36	$[M-H]^-$
hederagenin	$C_{30}H_{48}O_4$	472.36	$[M-H]^-$
Artanomaloide	$C_{32}H_{36}O_8$	548.24	$[M+H]^+$
Arteminolide B	$C_{35}H_{40}O_8$	588.27	$[M+H]^+$
Arteminolide C	$C_{35}H_{40}O_8$	588.27	$[M+H]^+$
Arteminolide A	$C_{35}H_{42}O_8$	590.29	$[M+H]^+$
Arteminolide F; Artenomaloide B	$C_{35}H_{42}O_8$	590.29	$[M+H]^+$

SI 5. Identification of phenolic acids from AaEA

Compounds	Formula	Molecular Weight (Da)	Ionization model
benzaldehyde	C_7H_6O	106.04	$[M+H]^+$
4-Methylphenol	C_7H_8O	108.06	$[M-H]^-$
benzoic Acid	$C_7H_6O_2$	122.04	$[M+H]^+$
4-Hydroxybenzaldehyde	$C_7H_6O_2$	122.04	$[M-H]^-$
1,3,5-Benzenetriol	$C_6H_6O_3$	126.03	$[M+H]^+$
4-Methoxybenzaldehyde	$C_8H_8O_2$	136.05	$[M+H]^+$
Phenyl acetate	$C_8H_8O_2$	136.05	$[M-H]^-$
4-Hydroxyacetophenone	$C_8H_8O_2$	136.05	$[M-H]^-$
Protocatechualdehyde	$C_7H_6O_3$	138.03	$[M-H]^-$
Salicylic acid	$C_7H_6O_3$	138.03	$[M-H]^-$
2,5-Dihydroxybenzaldehyde	$C_7H_6O_3$	138.03	$[M-H]^-$
4-Hydroxybenzoic Acid	$C_7H_6O_3$	138.03	$[M-H]^-$
Tyrosol	$C_8H_{10}O_2$	138.07	$[M-H]^-$
2-Nitrophenol	$C_6H_5NO_3$	139.03	$[M-H]^-$
Hydrocinnamic acid	$C_9H_{10}O_2$	150.07	$[M-H]^-$
4-AllylCatechol	$C_9H_{10}O_2$	150.17	$[M-H]^-$
Anisic acid	$C_8H_8O_3$	152.05	$[M+H]^+$
Vanillin	$C_8H_8O_3$	152.05	$[M-H]^-$
p-Hydroxyphenyl acetic acid	$C_8H_8O_3$	152.05	$[M-H]^-$
2,5-Dihydroxyacetophenone	$C_8H_8O_3$	152.15	$[M-H]^-$
2,3-Dihydroxybenzoic Acid	$C_7H_6O_4$	154.03	$[M-H]^-$
2,5-Dihydroxybenzoic Acid; Gentisic Acid	$C_7H_6O_4$	154.03	$[M-H]^-$

3,4-Dihydroxybenzoic Acid (Protocatechuic acid)	$C_7H_6O_4$	154.03	$[M-H]^-$
p-Coumaric acid	$C_9H_8O_3$	164.05	$[M+H]^+$
α-Hydroxycinnamic acid	$C_9H_8O_3$	164.05	$[M-H]^-$
(E)-3-(3,4-dihydroxyphenyl)acrylaldehyde	$C_9H_8O_3$	164.05	$[M+H]^+$
Ethyl phenylacetate	$C_{10}H_{12}O_2$	164.20	$[M+H]^+$
Ethylsalicylate	$C_9H_{10}O_3$	166.06	$[M+H]^+$
2-Hydroxy-3-phenylpropanoic acid	$C_9H_{10}O_3$	166.06	$[M-H]^-$
2,6-Dimethoxybenzaldehyde	$C_9H_{10}O_3$	166.06	$[M-H]^-$
3-(3-Hydroxyphenyl)-propionic acid	$C_9H_{10}O_3$	166.06	$[M-H]^-$
3-Hydroxyphenylacetic Acid Methyl Ester	$C_9H_{10}O_3$	166.06	$[M-H]^-$
3-(4-Hydroxyphenyl)-propionic acid	$C_9H_{10}O_3$	166.06	$[M-H]^-$
Rhododendrol	$C_{10}H_{14}O_2$	166.10	$[M+H]^+$
4-MethoxySalicylic acid	$C_8H_8O_4$	168.04	$[M-H]^-$
Gallacetophenone	$C_8H_8O_4$	168.04	$[M+H]^+$
Vanillic acid	$C_8H_8O_4$	168.04	$[M-H]^-$
5-MethoxySalicylic acid	$C_8H_8O_4$	168.15	$[M+H]^+$
Methyl 3,4-dihydroxybenzoate	$C_8H_8O_4$	168.15	$[M-H]^-$
Isovanillic acid	$C_8H_8O_4$	168.15	$[M+H]^+$
Gallic acid	$C_7H_6O_5$	170.02	$[M-H]^-$
Ethyl cinnamate	$C_{11}H_{12}O_2$	176.08	$[M+H]^+$
Coniferaldehyde	$C_{10}H_{10}O_3$	178.06	$[M+H]^+$
Caffeic acid	$C_9H_8O_4$	180.04	$[M-H]^-$
Syringaldehyde; 4-Hydroxy-3,5-Dimethoxybenzaldehyde	$C_9H_{10}O_4$	182.06	$[M-H]^-$
3-(3-Hydroxyphenyl)-3-hydroxypropanoic acid	$C_9H_{10}O_4$	182.06	$[M-H]^-$
Vanillic acid methyl ester	$C_9H_{10}O_4$	182.06	$[M-H]^-$

2,6-Dimethoxybenzoic Acid	$C_9H_{10}O_4$	182.17	$[M+H]^+$
Veratric acid	$C_9H_{10}O_4$	182.17	$[M+H]^+$
2,4-Dinitrophenol	$C_6H_4N_2O_5$	184.01	$[M-H]^-$
3-O-Methylgallic Acid	$C_8H_8O_5$	184.04	$[M-H]^-$
Methyl gallate	$C_8H_8O_5$	184.04	$[M-H]^-$
Ferulic acid	$C_{10}H_{10}O_4$	194.06	$[M-H]^-$
Methyl caffeate	$C_{10}H_{10}O_4$	194.06	$[M-H]^-$
Vanillin acetate	$C_{10}H_{10}O_4$	194.19	$[M+H]^+$
3,4-Dimethoxyphenyl acetic acid	$C_{10}H_{12}O_4$	196.07	$[M-H]^-$
Dihydroferulic Acid	$C_{10}H_{12}O_4$	196.07	$[M-H]^-$
syringic acid	$C_9H_{10}O_5$	198.05	$[M-H]^-$
2,6-Di-tert-butylphenol	$C_{14}H_{22}O$	206.17	$[M-H]^-$
Ferulic acid methyl ester	$C_{11}H_{12}O_4$	208.07	$[M-H]^-$
3,4-Dimethoxycinnamic acid	$C_{11}H_{12}O_4$	208.07	$[M-H]^-$
Ethyl caffeate	$C_{11}H_{12}O_4$	208.07	$[M-H]^-$
1-O-p-Cumaroylglycerol	$C_{12}H_{14}O_5$	238.08	$[M-H]^-$
3,4'-Dihydroxy-3'-methoxybenzenepentanoic acid	$C_{12}H_{16}O_5$	240.10	$[M-H]^-$
1-Feruloyl-sn-glycerol	$C_{13}H_{16}O_6$	268.10	$[M+H]^+$
p-Hydroxypheny-β-D-allopyranoside	$C_{12}H_{16}O_7$	272.09	$[M-H]^-$
α-Arbutin	$C_{12}H_{16}O_7$	272.25	$[M-H]^-$
cinnamoyltartaric acid	$C_{13}H_{12}O_7$	280.06	$[M-H]^-$
Phenethyl caffeate	$C_{17}H_{16}O_4$	284.11	$[M-H]^-$
1-O-Salicyl-D-glucose	$C_{13}H_{16}O_8$	300.08	$[M-H]^-$
4-O-Glucosyl-4-hydroxybenzoic Acid	$C_{13}H_{16}O_8$	300.08	$[M-H]^-$
Glucosyloxybenzoic Acid	$C_{13}H_{16}O_8$	300.09	$[M-H]^-$
Salidroside	$C_{14}H_{20}O_7$	300.12	$[M-H]^-$

Di-N-pentyl phthalate	$C_{18}H_{26}O_4$	306.18	[M−H]⁻
2-Acetyl-3-hydroxyphenyl-1-O-glucoside	$C_{15}H_{20}O_7$	312.12	[M−H]⁻
Protocatechuic acid-4-O-glucoside	$C_{13}H_{16}O_9$	316.08	[M−H]⁻
1-O-Gentisoyl-D-glucoside	$C_{13}H_{16}O_9$	316.08	[M−H]⁻
5-(2-Hydroxyethyl)-2-O-glucosylphenol	$C_{14}H_{20}O_8$	316.12	[M−H]⁻
p-Coumaric acid-4-O-glucoside	$C_{15}H_{18}O_8$	326.10	[M−H]⁻
1-O-p-Coumaroyl-β-D-glucose	$C_{15}H_{18}O_8$	326.10	[M−H]⁻
Phenylpropionic acid-O-β-D-glucopyranoside	$C_{15}H_{18}O_8$	326.10	[M−H]⁻
Raspberryketone glucoside	$C_{16}H_{22}O_7$	326.14	[M−H]⁻
Citrusin C	$C_{16}H_{22}O_7$	326.14	[M−H]⁻
Demethyl coniferin	$C_{15}H_{20}O_8$	328.12	[M−H]⁻
3-Hydroxy-4-isopropylbenzylalcohol-3-O-glucoside	$C_{16}H_{24}O_7$	328.15	[M−H]⁻
1-O-Vanilloyl-D-Glucose	$C_{14}H_{18}O_9$	330.10	[M−H]⁻
5-Glucosyloxy-2-Hydroxybenzoic Acid methyl ester	$C_{14}H_{18}O_9$	330.10	[M−H]⁻
5-O-Caffeoylshikimic acid	$C_{16}H_{16}O_8$	336.09	[M−H]⁻
3-O-p-Coumaroylquinic acid	$C_{16}H_{18}O_8$	338.10	[M−H]⁻
5-O-p-Coumaroylquinic acid	$C_{16}H_{18}O_8$	338.10	[M−H]⁻
Cichoriin	$C_{15}H_{16}O_9$	340.08	[M−H]⁻
1-O-Caffeoyl-β-D-glucose	$C_{15}H_{18}O_9$	342.10	[M−H]⁻
Vanillic Acid-4-O-Glucuronide	$C_{15}H_{18}O_9$	342.10	[M−H]⁻
coniferyl alcohol-4-O-glucoside (Coniferin)	$C_{16}H_{22}O_8$	342.13	[M−H]⁻
Cryptochlorogenic acid (4-O-caffeoylquinic acid)*	$C_{16}H_{18}O_9$	354.10	[M−H]⁻
Neochlorogenic acid (5-O-caffeoylquinic acid)	$C_{16}H_{18}O_9$	354.10	[M−H]⁻
Chlorogenic acid (3-O-caffeoylquinic acid)*	$C_{16}H_{18}O_9$	354.10	[M−H]⁻

4-O-Caffeoylquinic acid	$C_{16}H_{18}O_9$	354.10	$[M-H]^-$
1-caffeoylquinic acid	$C_{16}H_{18}O_9$	354.10	$[M-H]^-$
1-O-Feruloyl-β-D-glucose	$C_{16}H_{20}O_9$	356.11	$[M-H]^-$
Chlorogenic acid methyl ester	$C_{17}H_{20}O_9$	368.11	$[M-H]^-$
Sinapaldehyde-4-O-Glucoside	$C_{17}H_{22}O_9$	370.13	$[M+H]^+$
1-O-Sinapoyl-D-glucose	$C_{17}H_{22}O_{10}$	386.12	$[M-H]^-$
ethyl rosmarinate	$C_{20}H_{20}O_8$	388.12	$[M+H]^+$
5'-Glucosyloxyjasmanic acid	$C_{18}H_{28}O_9$	388.17	$[M-H]^-$
Benzyl β-primeveroside	$C_{18}H_{26}O_{10}$	402.15	$[M-H]^-$
Benzyl-(2''-O-xylosyl)glucoside	$C_{18}H_{26}O_{10}$	402.15	$[M-H]^-$
Salireposide	$C_{20}H_{22}O_9$	406.13	$[M+H]^+$
Robustaside B;[6'-O-Caffeoylarbutin]	$C_{21}H_{22}O_{10}$	434.12	$[M-H]^-$
6-O-Glucosyl-caffeoylbenzoic Acid	$C_{22}H_{22}O_{10}$	446.12	$[M-H]^-$
Caffeoyl-p-coumaroyltartaric acid	$C_{22}H_{18}O_{11}$	458.09	$[M-H]^-$
Dicaffeoylshikimic acid	$C_{25}H_{22}O_{11}$	498.12	$[M+H]^+$
3,6-Di-O-caffeoyl glucose	$C_{24}H_{24}O_{12}$	504.13	$[M-H]^-$
1,6-Di-O-caffeoyl-β-D-glucose	$C_{24}H_{24}O_{12}$	504.13	$[M-H]^-$
1-O-Caffeoyl-(6-O-glucosyl)-β-D-glucose	$C_{21}H_{28}O_{14}$	504.15	$[M-H]^-$
Isochlorogenic acid B	$C_{25}H_{24}O_{12}$	516.13	$[M-H]^-$
3,5-Dicaffeoylquinic acid	$C_{25}H_{24}O_{12}$	516.13	$[M-H]^-$
4,5-Dicaffeoylquinic acid	$C_{25}H_{24}O_{12}$	516.45	$[M+H]^+$
1,3-Dicaffeoylquinic acid	$C_{25}H_{24}O_{12}$	516.45	$[M+H]^+$
Rosmarinic acid-3'-O-glucoside	$C_{24}H_{26}O_{13}$	522.14	$[M-H]^-$
4,5-O-Dicaffeoylquinic acid Methyl Ester	$C_{26}H_{26}O_{12}$	530.14	$[M-H]^-$
3,5-O-Dicaffeoylquinic acid Methyl Ester	$C_{26}H_{26}O_{12}$	530.14	$[M+H]^+$
3,4-Di-O-caffeoylquinic acid methyl ester	$C_{26}H_{26}O_{12}$	530.50	$[M+H]^+$

3,4,5-Tricaffeoylquinic acid	$C_{34}H_{30}O_{15}$	678.16	$[M-H]^-$
Dicaffeoylquinic acid-O-glucoside	$C_{31}H_{34}O_{17}$	678.18	$[M-H]^-$
Syringoylcaffeoylquinic acid-D-glucose	$C_{31}H_{36}O_{18}$	696.19	$[M-H]^-$

SI 6. Identification of others from AaEA

Compounds	Formula	Molecular Weight (Da)	Ionization model
2-Ethylpyrazine	$C_6H_8N_2$	108.07	$[M+H]^+$
3-Methylbenzaldehyde	C_8H_8O	120.06	$[M-H]^-$
4-Guanidinobutanal	$C_5H_{11}N_3O$	129.09	$[M+H]^+$
5,7-Dihydroxychromone	$C_9H_6O_4$	178.03	$[M-H]^-$
Eucommiol	$C_9H_{16}O_4$	188.11	$[M-H]^-$
2,5-dimethyl-7-hydroxy-chromone	$C_{11}H_{10}O_3$	190.06	$[M-H]^-$
Butylphthalide	$C_{12}H_{14}O_2$	190.24	$[M+H]^+$
N,N'-dicyclohexylcarbodiimide	$C_{13}H_{22}N_2$	206.18	$[M-H]^-$
2,4'-Dihydroxybenzophenone	$C_{13}H_{10}O_3$	214.22	$[M+H]^+$
2-methyl-5-carboxy methyl-7-Hydroxy-chromone	$C_{12}H_{10}O_5$	234.05	$[M-H]^-$
Vidarabine	$C_{10}H_{13}N_5O_4$	267.10	$[M+H]^+$
Ranunculinin glucoside(iso-01)	$C_{11}H_{16}O_8$	276.08	$[M+H]^+$
6-demethoxycapillarisin	$C_{15}H_{10}O_6$	286.05	$[M+H]^+$
Trans dehydrorosinone	$C_{19}H_{28}O_2$	288.21	$[M-H]^-$
3,5,7,4'-Tetrahydroxy-Coumaronochromone	$C_{15}H_{10}O_7$	302.04	$[M+H]^+$
capillarisin	$C_{16}H_{12}O_7$	316.06	$[M+H]^+$
N-benzoyl-2-aminoethyl-β-D-glucopyranoside	$C_{15}H_{21}NO_7$	327.13	$[M+H]^+$
Bartsioside	$C_{15}H_{22}O_8$	330.13	$[M+H]^+$
α-Conidendrin	$C_{20}H_{20}O_6$	356.13	$[M+H]^+$
4-Ketopinoresinol	$C_{20}H_{20}O_7$	372.12	$[M-H]^-$
Dehydrodiconiferyl alcohol-4-O-glucoside	$C_{26}H_{32}O_{11}$	520.19	$[M+H]^+$

SI 7. The primers used for Real-time PCR analysis

Genes	Forward primer sequences, 5’-3’	Reverse primer sequences, 5’-3’
β-Actin	GTGCTATGTTGCTCTAGACTTCG	ATGCCACAGGATTCCATACC
TLR4	AATGAGGACTGGGTGAGAAATGAGC	TCCTGGATGATGTTGGCAGCAATG
MyD88	TCTCAATTAGCTCGCTGGCAATGG	GATCCGGCAACTAGAACAGACAGAC
NF-κB p65	CTGAAGCTATAACTCGCCTGGTGAC	CTAGTCCGCAATGGAGGAGAAGTC
IKK	ATGGACAGGCGTTGATGCTTCG	GGCTGCTGCTGAGGTAGAGGAG
IκB α	GAGGAGTACGAGCAAATGGTGAAGG	GCCAAGTGCAGGAACGAGTCTC
Bcl2	CCGTCGTGACTTCGCAGAGATG	ATCCCTGAAGAGTTCCTCCACCAC
Bax	CGTGAGCGGCTGCTTGTCTG	ATGGTGAGCGAGGCGGTGAG

SI 8. Schematic diagram of regulating Bax/Bcl-2 and TLR4/MyD88/NF-κB pathways in the treatment of the immune liver injury.

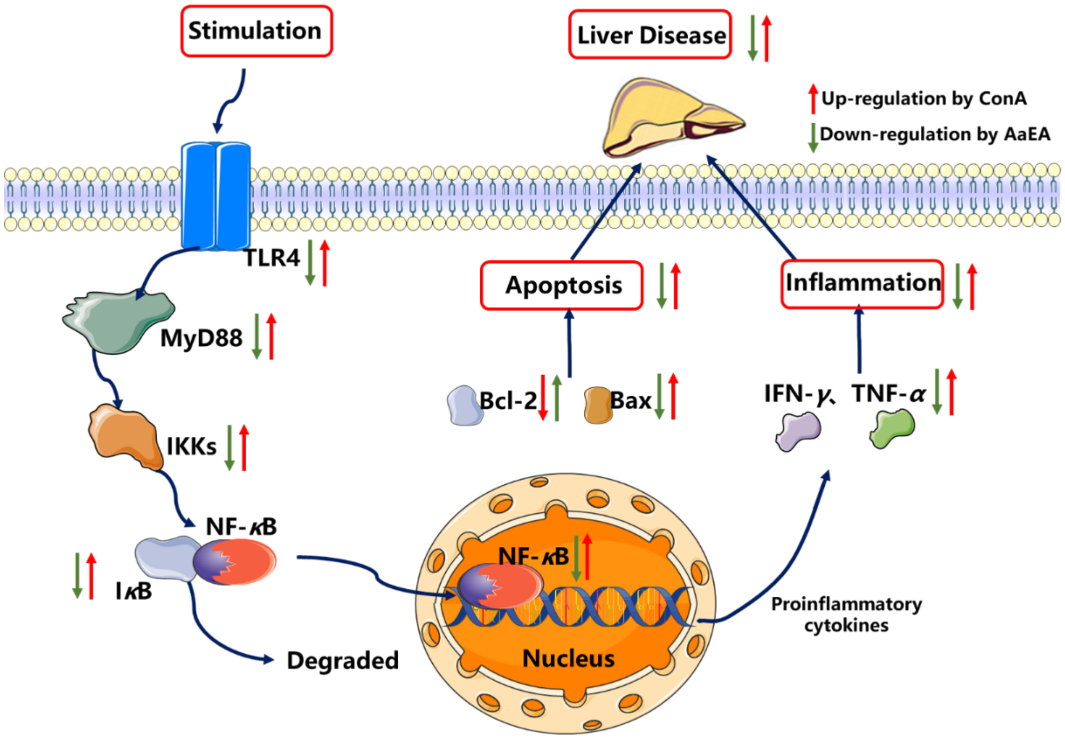

SI 9. The original images of Western Blot.

The relative key proteins we measured by Western Blotting analysis were shown as follows. There were some reasons that the bands had to be separated to incubated with primary antibodies and then detected, respectively. First, there were some proteins with the same weight or their weights were very close, for instance, IKK and p-IKK as well as I*κ*B and p-I*κ*B, so they can not be detected at the same time. Second, different proteins require different times to expose by the instrument. Third, due to limited funds, the separated bands to incubate with primary antibodies respectively were more suitable.

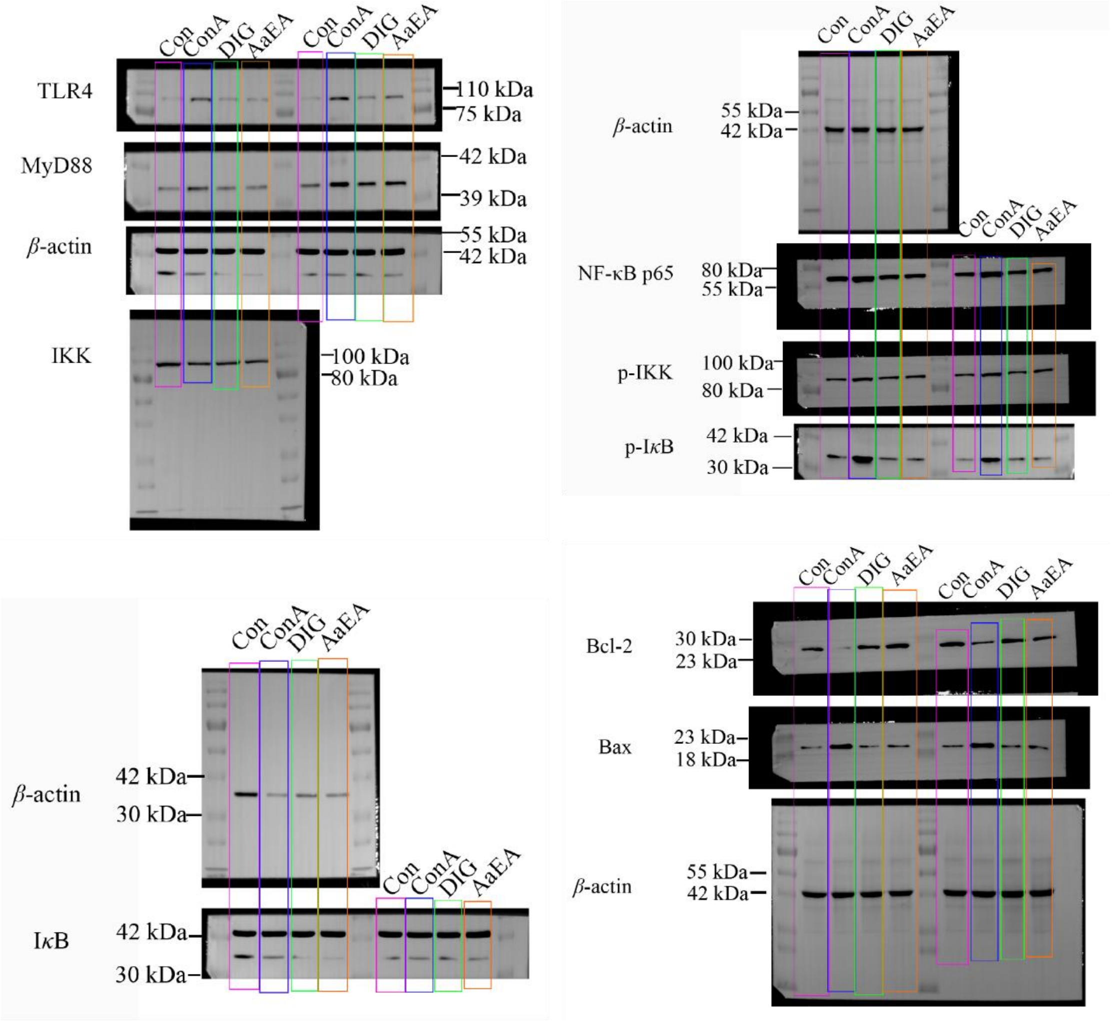

Supplement: Supplementary file 1 [file molecules-27-07883-s001.zip › molecules-2021785-supplementary.pdf]
